# The global and promoter-centric 3D genome organization temporally resolved during a circadian cycle

**DOI:** 10.1186/s13059-021-02374-3

**Published:** 2021-06-08

**Authors:** Mayra Furlan-Magaril, Masami Ando-Kuri, Rodrigo G. Arzate-Mejía, Jörg Morf, Jonathan Cairns, Abraham Román-Figueroa, Luis Tenorio-Hernández, A. César Poot-Hernández, Simon Andrews, Csilla Várnai, Boo Virk, Steven W. Wingett, Peter Fraser

**Affiliations:** 1 Departamento de Genética Molecular, Instituto de Fisiología Celular, Universidad Nacional Autónoma de México, 04510 Mexico City, Mexico; 2 Cancer Research UK Cambridge Institute, University of Cambridge, Cambridge, CB2 0RE UK; 3 Laboratory of Neuroepigenetics, Medical Faculty of the University of Zurich and Department of Health Science and Technology of the Swiss Federal Institute of Technology, Neuroscience Center Zurich, Zurich, Switzerland; 4 Nuclear Dynamics Programme, The Babraham Institute, Cambridge, CB22 3AT UK; 5 Wellcome-MRC Cambridge Stem Cell Institute, Jeffrey Cheah Biomedical Centre, Cambridge, CB2 0AW UK; 6 Unidad de Bioinformática y Manejo de Información, Instituto de Fisiología Celular, Universidad Nacional Autónoma de México, 04510 Mexico City, Mexico; 7 Bioinformatics Group, The Babraham Institute, Cambridge, CB22 3AT UK; 8 Centre for Computational Biology, University of Birmingham, Birmingham, B15 2FG UK; 9 Institute of Cancer and Genomic Sciences, University of Birmingham, Birmingham, B15 2SY UK; 10 Cell Biology Division, MRC Laboratory of Molecular Biology, Francis Crick Avenue, Cambridge Biomedical Campus, Cambridge, CB2 0QH UK; 11 Department of Biological Science, Florida State University, Tallahassee, FL USA

**Keywords:** Genome 3D organization, Promoter interactions, Circadian gene expression, Transcription regulation, TADs, Chromatin compartments, Enhancers, Circadian rhythms

## Abstract

**Background:**

Circadian gene expression is essential for organisms to adjust their physiology and anticipate daily changes in the environment. The molecular mechanisms controlling circadian gene transcription are still under investigation. In particular, how chromatin conformation at different genomic scales and regulatory elements impact rhythmic gene expression has been poorly characterized.

**Results:**

Here we measure changes in the spatial chromatin conformation in mouse liver using genome-wide and promoter-capture Hi-C alongside daily oscillations in gene transcription. We find topologically associating domains harboring circadian genes that switch assignments between the transcriptionally active and inactive compartment at different hours of the day, while their boundaries stably maintain their structure over time. To study chromatin contacts of promoters at high resolution over time, we apply promoter capture Hi-C. We find circadian gene promoters displayed a maximal number of chromatin contacts at the time of their peak transcriptional output. Furthermore, circadian genes, as well as contacted and transcribed regulatory elements, reach maximal expression at the same timepoints. Anchor sites of circadian gene promoter loops are enriched in DNA binding sites for liver nuclear receptors and other transcription factors, some exclusively present in either rhythmic or stable contacts. Finally, by comparing the interaction profiles between core clock and output circadian genes, we show that core clock interactomes are more dynamic compared to output circadian genes.

**Conclusion:**

Our results identify chromatin conformation dynamics at different scales that parallel oscillatory gene expression and characterize the repertoire of regulatory elements that control circadian gene transcription through rhythmic or stable chromatin configurations.

**Supplementary Information:**

The online version contains supplementary material available at 10.1186/s13059-021-02374-3.

## Background

Circadian variation of gene expression in the liver is essential to temporally coordinate metabolic processes including lipid and glycogen metabolism, and to maintain organism homeostasis. Considerable progress has been made in our understanding of the transcription factors that control circadian transcription regulation [1, 2]. However, the impact of 3D chromatin configuration dynamics over the course of a day in circadian oscillations of gene expression is still poorly understood.

Previous work in cultured cells looking at chromatin contacts established by specific genomic loci showed that the circadian gene *Dbp* forms inter-chromosomal contacts with ~ 200 kb genome blocks, with fluctuating strength over the course of a day in cultured cells [3]. At higher resolution, enhancer-promoter contacts of the core-clock gene *Cry1* and clock output genes *Mreg*, *Slc45a3*, *Gys2* have been shown to oscillate daily in a *Arntl*-dependent manner in liver [4–6]. In contrast, analysis of Arntl cistrome showed that Arntl occupied regulatory elements, establishing stable contacts during the day [7]. Also the *Nr1d1* circadian gene forms invariant contacts throughout the circadian cycle to a nearby super-enhancer with the help of Cohesin [8]. Finally, genome-wide Hi-C studies at two timepoints of a day-night cycle suggested that circadian targeted by Nr1d1 repressor protein form contacts within their respective topologically associating domains (TADs) that can be dynamic over time [9].

Despite evidence of dynamic and stable contacts established by candidate circadian genes, we still lack an understanding of what factors distinguish rhythmic and constant genomic contacts formed by circadian genes with maxima of transcriptional output (acrophases) at different times, and how common these types of chromatin contacts are when analyzing all the circadian gene promoters in the genome. Also, analysis of a high-resolution genome-wide promoter centric panorama of all circadian contacts resolved in time is required to answer these questions. Here we present results from in-nucleus Hi-C and Promoter-Capture Hi-C (P-CHi-C) at 4 timepoints during a day in mouse adult liver. We provide for the first time a temporally resolved genome-wide contact analysis at different scales, encompassing genomic compartments (A, B compartments), mega to kilo-base domains (TADs), and high-resolution coverage of contacts from all individual gene promoters including circadian gene promoters. We identify instances of genomic A/B compartment assignment changes in parallel to circadian modulation of histone modifications and oscillatory gene expression. Many of the circadian genes that changed A/B assignment are found in TADs that remain constant during the day. Analysis of gene promoter interactions at restriction fragment resolution through P-CHi-C, revealed that circadian gene promoters form dynamic and constant contacts, increasing the number of genomic contacts at the acrophase of the corresponding transcriptional units. Furthermore, we found liver nuclear receptors (e.g., Nr5a2) binding motifs enriched at both dynamic and constant circadian contacting regions and other transcription factor (TF) binding motifs unique for dynamic or constant promoter contacts (e.g., immediate early factors Tcfap2 and Fos:Jun). Furthermore, diurnal and nocturnal circadian gene promoters preferentially contacted enhancers and other circadian promoters transcriptionally active during day and night time, respectively. Finally we found that core clock-associated gene promoters engage in more dynamic interactomes than clock output circadian genes in the liver. Our detailed analysis of the 3D map of the gene promoter interactome over a 24-h cycle in the liver indicates that conformation dynamics at different genomic scales are coupled with circadian transcriptional oscillations.

## Results

### Circadian A/B chromatin compartments switch between open and closed configurations throughout the day

To study global genome architecture during a circadian cycle, we performed in-nucleus Hi-C (see “Methods”) on mouse adult liver at four different timepoints of the circadian cycle (ZT0, 6, 12 and 18), with ZT0 and ZT12 being the start of the light and dark phase, respectively, in three biological replicates. Samples of individual livers were processed in parallel for RNA-seq. We produced high-quality Hi-C data sets with a high percentage of valid pairs (~ 80%), low PCR duplicates (less than 2%), and high cis:trans interaction ratios obtained (~ 80:20%) (Table S1). In total, we obtained ~ 2 billion valid Hi-C read pairs from mouse adult liver across a circadian cycle (Table S1).

**Fig. 1:**
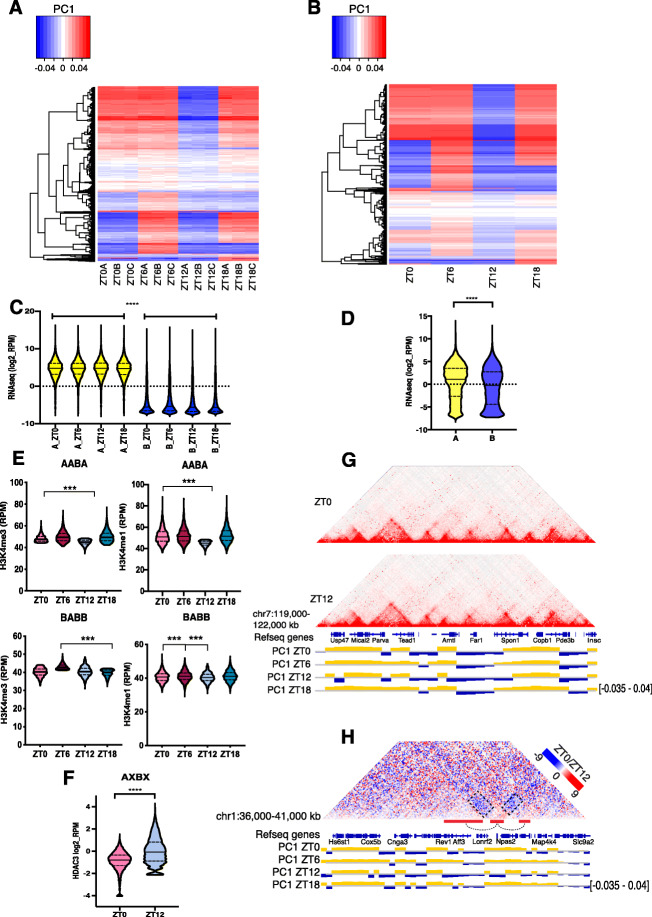
Chromatin compartments change during the 24 h. **a** Heatmap of PC1 values for OCCs (independent replicates, significant variance *p* value < 2e−16, one-way ANOVA). **b** Heatmap of PC1 values for OCCs (merged replicates, significant variance *p* value < 2e−16, one-way ANOVA). **c** RNA-seq reads coverage (log2_RPM) in all A vs all B compartments across timepoints during the circadian cycle (*p* < 0.0001, one-way ANOVA test). **d** RNA-seq reads coverage (log2_RPM) in OCCs A vs B compartments (*p* < 0.0001, Mann-Whitney test). **e** H3K4me3 and H3K4me1 RPM ChIP-seq signal in OCCs across timepoints. BABB (ZT0 = B, ZT6 = A, ZT12 = B, ZT18 = B) and AABA (ZT0 = A, ZT6 = A, ZT12 = B, ZT18 = A) (all *p* values < 0.001, one-way ANOVA test, Tukey post hoc test). **f** HDAC3 ChIPseq log2_RPM signal in OCCs at ZT0 and ZT12 (*p* value < 0.0001, Wilcoxon rank test). **g** Observed Hi-C contacts from ZT0 and ZT12 liver samples at the *Antl1* circadian gene TAD genomic region. PC1 values plotted underneath. *Arntl* cTAD switches chromatin compartment at ZT12. H. ZT0/ZT12 differential Hi-C contact matrix of a region including the *Npas2* circadian gene TAD switching chromatin compartment at ZT12. PC1 values plotted underneath

To detect “open,” transcriptionally active and “closed,” silent genomic compartments (A and B compartments, respectively), we performed PCA analysis on Hi-C data at different timepoints throughout the circadian cycle, at 100-kb bin resolution. PCA analysis is used to analyze high dimensional data and we redefine them, with as few dimensions as possible that explain most of the variance in the data. As such, the 1st principal component (PC1) will explain the majority of the variance, followed by the second component (PC2) and so forth. When applying PCA to the normalized contact Hi-C matrix from individual chromosomes, important features can be identified. For most chromosomes, the PC1 value reflects two distinct interaction compartments that correspond to open and closed chromatin [10]. Changes in chromatin compartments have been associated with changes in transcription and chromatin states during cell differentiation and mouse early development [11, 12]. As expected, PC1 values partitioned the liver genome into chromatin compartments (Fig. 1a,b, Additional file 1: Figure S1A,B). We then compared the eigenvectors of the different timepoints and identified changes in the sign of regional PC1 values, indicative of compartment switching between all timepoint pairs (Fig. 1a,b, individual replicates and merged replicates, respectively, Additional file 1: Figure S1C one-way ANOVA *p* value < 2e − 16). These genomic regions, termed oscillatory chromatin compartments (OCCs) spanned 440.4 Mb of the mouse genome. The rest of the genome (82.7%) retained the same compartment identity during the 24-h cycle (Additional file 1: Figure S1A,B individual replicates and merged replicates, respectively, S1D). We found OCCs with compartment assignments ZT0 = A, ZT6 = A, ZT12 = B, ZT18 = A (AABA) being the most abundant type in the genome covering 194.7 Mb (Additional file 1: Figure S1E).

To relate chromatin compartments with transcription, we performed stranded total RNA-seq. We obtained ~ 500,000,000 of 150-bp reads per timepoint (4 biological replicates each) for a total of ~ 2,000,000,000 reads (Table S2). Spearman correlation analysis showed good correlation between the 4 biological replicates (Additional file 2: Figure S2A, ZT0 and 12 shown). Genes with differential expression between at least one pair of timepoints were identified (*q*-value < 0.01) and classified as circadian. In total, we detected 1257 circadian gene transcripts (Additional file 2: Figure S2B, Table S3). Inspection of individual gene expression profiles from our RNA-seq showed the expected oscillatory expression pattern for examples of both core-clock and output circadian genes in the liver (Additional file 2: Figure S2C,F). Gene Ontology and KEGG pathway analysis identified circadian rhythm and metabolism as significantly enriched categories in our identified circadian gene set (Additional file 2: Figure S2D,E) confirming efficient detection of circadian oscillating genes. RT-qPCR of primary and mature mRNAs confirmed oscillation of circadian gene expression (Additional file 2: Figure S2G). Analysis of the RNA content in A and B compartments at all timepoints revealed that A compartments are RNA-rich and B are RNA-poor (Fig. 1c, *p* < 0.0001, one-way ANOVA test). The RNA content in OCCs falling into A or B at different times of the day was also significantly different (Fig. 1d, *p* < 0.0001, Mann-Whitney test). In addition to RNA abundance, we analyzed time resolved enrichment of histone modifications characteristic of open chromatin H3K4me3 and H3K4me1 [13] in OCCs around the clock. Both histone marks were significantly enriched in regions at times of A compartment assignment compared to B compartment across all timepoints (Fig. 1e, AABA [ZT0 = A, ZT6 = A, ZT12 = B, ZT18 = A] and BABB [ZT0 = B, ZT6 = A, ZT12 = B, ZT18 = B], Additional file 1: Figure S1F, AABB, ABBB, BABA. All *p* values < 0.001, one-way ANOVA test, Tukey post hoc test). Histone deacetylase 3 (HDAC3) binding to chromatin is enriched in deacetylated “closed” chromatin and it has been measured in mouse liver at ZT22 and ZT10 [14]. We quantified HDAC3 occupancy at OCCs at ZT0 and 12 (our closest timepoints to ZT22 and 10). HDAC3 binding was higher at OCCs that fell into the B compartment at ZT12 compared to the A compartment at ZT0 (Fig. 1f, Wilcoxon *p* < 0.001). Examples of OCCs overlapping the circadian *Arntl* gene TAD and the circadian *Npas2* gene TAD are shown in Fig. 1g and h respectively. For all circadian genes presented as examples throughout the paper, the RNA-seq signal tracks for the four timepoints examined can be found in Additional file 3: Figure S3. Together, these results show that both transcription and chromatin state fluctuate in accordance with compartment switching during a circadian cycle.

### Topologically associated domains spatially partition temporal gene expression control but remain structurally invariant during a circadian cycle

**Fig. 2:**
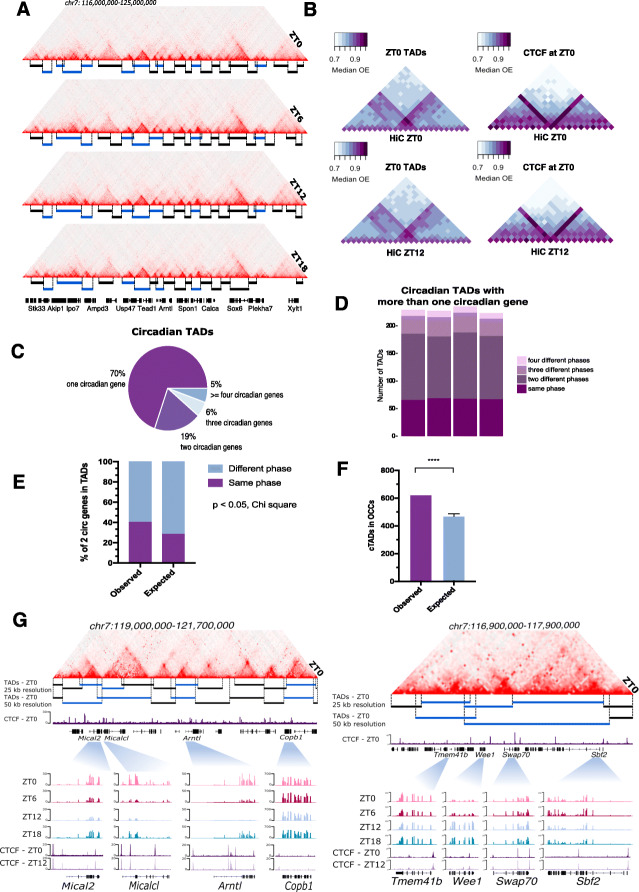
Circadian TADs isolate circadian genes with shared time of transcription. **a** Observed Hi-C contact matrices showing the TADs landscape of the genomic region including the *Arntl1* circadian gene locus at four timepoints during the circadian cycle. **b** Left, 50 kb resolution median observed/expected ZT0 and ZT12 Hi-C signal around 1000 randomly selected TADs from ZT0 plotted on ZT0 and ZT12 Hi-Cs. TADs were scaled to fit the five central bins. Right, the same metaplots but for 1000 randomly CTCF peaks found at ZT0. CTCF peaks are at the central bin of the metaplot. **c** Proportion of cTADs harboring 1, 2, 3, or 4 circadian genes. **d** Phase distribution of circadian genes sharing TADs. **e** Observed and expected proportion of TADs with 2 circadian genes sharing transcriptional peak phase (*p* value < 0.05, chi-square test). **f** Observed and expected number of circadian TADs overlapping and non-overlapping OCCs (Wilcoxon test, *p* < 0.0001). **g** Examples of cTAD Hi-C contact matrices from ZT0. Left, cTADs with one circadian gene allocating *Mical2, Micalcl, Arntl1*, and *Copb1* circadian genes. Right, cTADs with more than one circadian gene allocating *Tmem41b, Wee1, Swap70*, and *Sbf2* circadian genes. Close up to each circadian gene with genomic tracks showing RNA-seq signal at all timepoints and CTCF ChIP-seq peaks at ZT0 and ZT12

To identify TADs, we assigned TAD insulation scores to the Hi-C contact matrices [15] and examined them across timepoints (see “Methods”). TADs displayed little variation across timepoints as has been previously observed [9]. Of the total of 4358 TADs that we identified, 2936 were preserved throughout the day and 952 at least between two timepoints and presented a size distribution between 150 kb and 1.5 Mb (Fig. 2a and Additional file 4: Figure S4A,B). To confirm insulation of TADs across timepoints, we selected a random set of 1000 TADs detected at ZT0 and plotted their median observed/expected contacts using Hi-C data from ZT0 and ZT12 (see “Methods”). We recovered higher contact frequencies within than outside TADs at both timepoints confirming preservation of domain structures across timepoints (Fig. 2b left panel). The same result held true for TADs detected at ZT6, 12 and 18 h (Additional file 4: Figure S4C).

CTCF functions as an architectural protein that establishes chromatin domains together with cohesin in mammalian chromatin [16–18]. We performed ChIP-seq against CTCF at ZT0 and ZT12 on the same liver samples used for Hi-C. In total, 33,262 CTCF binding sites (75.3%), out of a total of 44163 sites, were shared between ZT0 and 12 and CTCF showed similar enrichment between the two timepoints (Additional file 4: Figure S4D). When plotting the Hi-C contacts in an aggregate peak analysis centered on the CTCF binding sites, CTCF-bound regions exhibited robust contact insulation properties, independent of the timepoint examined (Fig. 2b right panel and Additional file 4: Figure S4E top panels). Additionally, we assessed preservation of CTCF insulation between mouse ES cells and liver cells by overlaying regions occupied by CTCF in mESCs [19], onto our liver Hi-C data sets. Robust insulation was observed when using either ZT0 or ZT12 Hi-C data suggesting large agreement of CTCF chromatin occupancy and insulation properties between mESCs and adult liver tissue (Additional file 4: Figure S4 E bottom panels). The conservation of CTCF binding between ESCs and adult liver tissue shows that the insulation of circadian genes by CTCF precedes the onset of their rhythmic transcription, as ESCs do not harbor functional circadian oscillators [20].

Next, we assigned genes to TADs. We found that on average TADs harbored 7.2 genes. TADs containing one or more circadian genes (cTADs) were larger than non cTADs and on average contained more genes (14.2) (Additional file 4: Figure S4F, all *p* values < 2.2e−16, Wilcoxon rank sum test). We observed that 70% of cTADs contained only one circadian gene (Fig. 2c). The remaining cTADs contained more than one circadian gene and remarkably, the circadian genes sharing TADs exhibited peak transcriptional expression at shared times during the day (40% for TADs with 2 circadian genes compared to the expected 28%, 18% of TADs with 3 circadian genes compared to the expected 8.8%, *p* < 0.0001, chi square test) (Fig. 2d and e, only data from cTADs with 2 circadian genes shown). We next analyzed whether TADs encoding circadian genes switched chromatin compartment over the circadian cycle. Indeed, most cTADs (73%, 623) overlapped with OCCs more than expected when compared to a random set of the same number of non-cTADs (Wilcoxon test, *p* < 0.0001) (Fig. 2f). Examples of cTADs containing one circadian gene (cTADs with *Mical2*, *MicalcI*, *Arntl* and *Copb1*, respectively), or more than one circadian gene (cTAD with *Wee1* and *Swap70* genes, both with acrophase at ZT12) are shown in Fig. 2g (left and right panels respectively). Examples of cTADs overlapping OCCs are shown for *Arntl* (Fig. 1g) and *Npas2* (Fig. 1h) as previously described. These results show that cTADs often set transcriptional phase coherence between multiple circadian genes in the same TAD. While most TADs maintain their structural boundaries over time, they overlap with compartments that switch between active and silent states throughout a circadian cycle.

### Gene promoter-promoter interactions in the liver and its circadian component

**Fig. 3:**
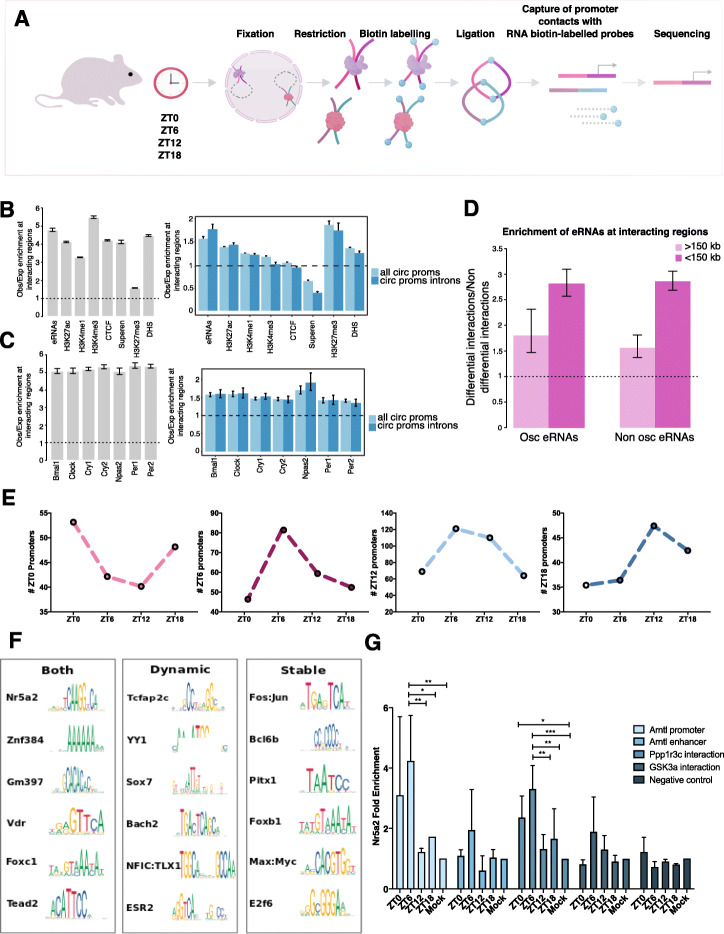
Promoter Capture-Hi-C and chromatin contact dynamics during a circadian cycle. **a** Summary of the experimental workflow of Promoter-CHi-C technology. Cells are fixed, chromatin is digested, filled-in, and biotin-labeled inside the nucleus. Pull down with streptavidin beads is then performed and Hi-C libraries prepared for sequencing. Using the Hi-C material as a template hybiridization is done using the designed RNA biotinilated probes to capture promoters. A second pull down is performed to recover the hybrid molecules, DNA purified and sequenced. **b** Left, Obs/Exp signal ratio at promoter interacting regions of liver chromatin features including enhancers producing eRNAs, H3K27ac, H3K4me1, H3K4me3, DNase I hypersensitive sites, superenhancers, H3K27me3, and CTCF (left) (all *p* values < 8.632642e−123, *t* test). Obs/Exp signal ratio of the same chromatin features but for interacting regions for all oscillating gene promoters and intronically oscillating gene promoters compared to a random set of non-oscillating gene promoters (all *p* values < 2.525639e−42 except H3K27me3). **c** Right, the same as in **d** but for the enrichment of circadian transcription factors including Bmal1, Clock, Cry1, Cry2, Npas2, Per1, and Per2 for all gene promoter interacting regions and left, oscillatory gene promoter interacting regions (all *p* values < pval< 3.870992e−205, *t* test). **e** Obs/Exp enrichment of enhancers producing eRNAs at dynamic contacts over stable contacts (*p* value < 2e−16, *t*-test). **f** Number of circadian gene promoters making the maximum number of contacts at ZT0, 6, 12, and 18 (*p* value < 0.001, chi-square test). **g** Transcription factor DNA binding motifs significantly enriched at dynamic, stable, or both circadian gene promoter chromatin contacts (*E*-value < 1.00e−002). **g** ChIP-qPCR against Nr5a2. The regions analyzed include the *Arntl* gene promoter, the *Arntl* dynamically interacting enhancer, a constant interacting element of the *Pppr1r3c* circadian gene, and a dynamic interacting element of the *Gsk3a* circadian gene. In all regions, the DNA binding motif of Nr5a2 was found. As a negative control, a region where the DNA binding motif for Nr5a2 is not present was amplified (*p value < 0.05, ** *p* value 0.01, *** *p* value < 0.001, one-way ANOVA, Tukey post hoc test, *n* = 6 from 2 biological replicates per timepoint)

To gain insights into chromatin contacts at the level of individual circadian genes, we measured genome-wide promoter-promoter and promoter-regulatory element contacts at four timepoints during a circadian cycle using Promoter-CHi-C (Fig. 3a) [21–23]. Promoter-containing ligation products from Hi-C libraries were efficiently captured (~ 70%) using 39,021 RNA probes, which hybridize to 22,225 genomic restriction fragments covering all annotated gene promoters in the mouse genome. We produced ~ 1,560,000,000 total valid read pairs from the three biological replicates for the four timepoints, thus obtaining ~ 390,000,000 valid ligation products per timepoint (Table S4). Capture of gene promoters increased the number of valid ligation products per promoter to ~ 10–15 fold compared to Hi-C. An example of this enrichment is shown for the *Arntl* gene locus comparing a virtual 4C from the *Arntl* gene promoter performed on the Hi-C data versus the Promoter-CHi-C chromatin contact data sequenced at equivalent depth (Additional file 5: Figure S5A and S5B comparison of raw paired reads per restriction fragment in CHi-C vs Hi-C from the virtual 4C from the *Arntl* gene promoter *p* < 0.0001, Wilcoxon rank test). We estimated the statistical significance of interactions between pairs of promoters and between promoter and other, potentially gene regulatory genomic regions, using the CHiCAGO pipeline for each timepoint (“Methods”) [24]. We obtained ~ 150,000 statistically significant interactions per timepoint resulting in ~ 600,000 statistically significant promoter interactions in total (Table S4).

First, we focused on gene promoter-promoter contacts. We built promoter-promoter networks at all timepoints and found large disconnected networks as expected for promoters scattered across the different chromosomes (Additional file 5: Figure S5C, ZT0 shown). We then evaluated the larger clusters of the promoter-promoter network containing hundreds of connected gene promoters (Additional file 5: Figure S5D, the 4 larger clusters shown including inter- and intrachromosomal promoter-promoter interactions) and performed gene enrichment analysis on these using gProfiler [25] (Additional file 5: Figure S5E). We found the Major Histocompatibility Complex forming one cluster with genes encoded on chromosomes 7 and 17. MHC genes are lowly expressed in the healthy liver and constitute dense, highly connected chromatin [26] (Additional file 5: Figure S5D and S5E, cluster 1). Similarly, a transcriptionally repressed cluster is formed between olfactory receptor genes on chromosome 7 (Additional file 5: Figure S5D and S5E, cluster 3). In addition to repressed genes clustering in spatial proximity, we identified actively transcribed genes involved in glutathione synthesis and amino acid metabolism essential for liver detoxification function arranged in a promoter network (Additional file 5: Figure S5D and S5E, cluster 2). Another cluster encompassed constitutive histone genes located in chromosomes 3, 13, 11, 18, 12, and 7, among others (Additional file 5: Figure S5D and S5E, cluster 4). Thus, prominent constitutive and liver-specific promoter-promoter networks both transcriptionally active and inactive were identified in the adult liver.

Next we examined the circadian component in promoter-promoter networks. Circadian promoters are dispersed across chromosomes (Additional file 6: Figure S6A, blue dots represent circadian gene promoters). Nevertheless, circadian promoters establish significantly more contacts among them compared to non-circadian gene promoters (Additional file 6: Figure S6B. Above, comparison of the number of edges formed between circadian promoters compared to a random set of non-circadian gene promoters. Below, *Z*-scores compared to the random sampling of non-circadian promoters). Next we looked at the reads supporting significant interactions between circadian gene promoters in contrasts with interactions between non circadian gene promoters. The result shows that circadian promoter-promoter contacts are more robust compared to non-circadian promoter-promoter contacts (Additional file 6: Figure S6C, *p* values < 0.001, Mann-Whitney test). Finally, we compared the time of maximal mRNA abundance of circadian genes whose promoters were contacting each other. To do this, we separated the circadian gene promoters into diurnal and nocturnal based on their transcriptional acrophase and then analyzed the time of maximal RNA expression of their contacted circadian gene promoters. We found that diurnal promoters preferentially contacted other diurnal genes. Likewise, nocturnal circadian genes preferentially contacted other genes with nocturnal expression maxima (Additional file 6: Figure S6D, our intronic circadian gene set, *p* values < 0.0001 Wilcoxon signed rank test). Similar results were obtained for circadian genes detected by GROseq [27] reflecting primary transcription (Additional file 6: Figure S6E, *p* values < 0.0001 Wilcoxon signed rank test). This is exemplified by the interaction between the *Tef* and the *Aco* circadian gene promoters with shared pre-messenger expression peak at ZT12 (Additional file 6: Figure S6F) as well as the interaction between *Rorc* and *Cgn* circadian genes with shared acrophase at ZT18 (Additional file 7: Figure S7A). Thus, in summary the results show that circadian promoter-promoter contacts are more robust than non-circadian promoter-promoter contacts. Furthermore, pairs of interacting circadian gene promoters tend to share their transcriptional acrophase.

### Regulatory elements form dynamic and stable chromatin contacts with circadian gene promoters

In order to characterize the full range of promoter interactions, we next examined contacts between promoters and non-promoter genomic regions. Genomic regions that interact with gene promoters including circadian gene promoters in mouse liver, were enriched for histone modifications characteristic of regulatory elements such as H3K27ac, H3K4me1, H3K4me3, and H3K27me3, as well as DNase I hypersensitive sites and the structural protein CTCF [13, 28, 29], compared to distance matched non-interacting regions (Fig. 3b left panel, all *p* values < 8e−166, *t* test, CTCF, *p* value <8e−18; *t* test). A set of enhancers from which enhancer RNAs (eRNAs) are produced have been described in the liver [27]. We found that these enhancers were enriched at the contacted regions (Fig. 3b, left panel *p* value < 9e−165; *t* test). Overall, the chromatin features at promoter-contacting regions suggest that our P-CHi-C captured potential structural and/or regulatory elements.

**Fig. 4:**
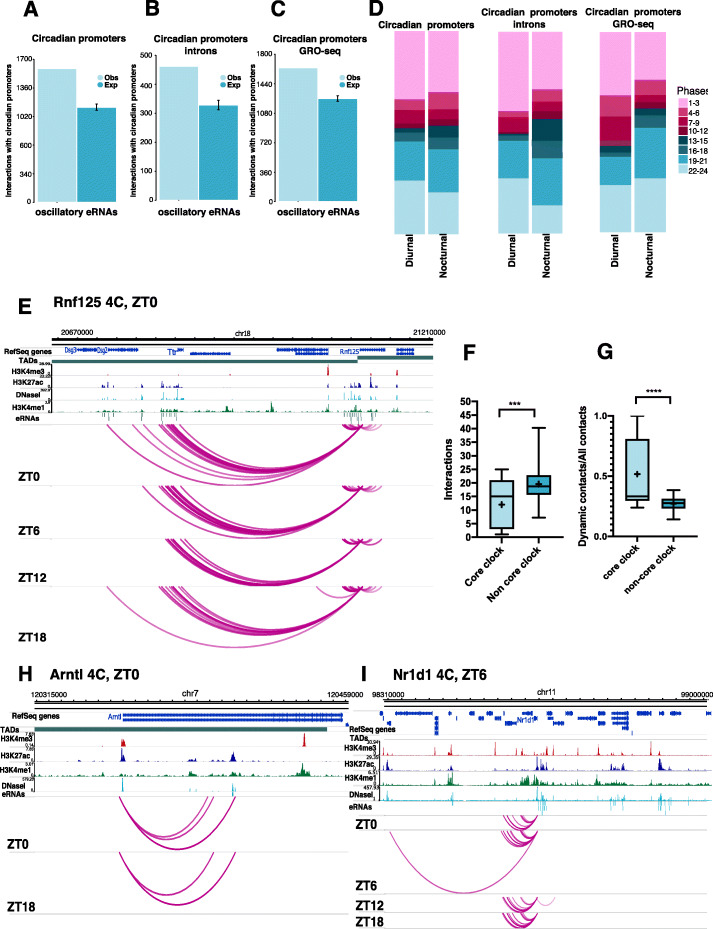
Transcriptional phase coherence between circadian genes and transcribed enhancers and the core-clock circadian genes display highly dynamic chromatin contacts. **a** Circadian gene promoter observed and expected contacts with enhancers producing eRNA rhythmically during the day (*p* value < 0.0001, *t* test). **b** Circadian genes oscillating at the intronic level observed and expected contacts with enhancers producing eRNAs rhythmically during the day (*p* value < 0.0001, *t* test). **c** Observed and expected contacts of circadian gene promoters detected through GRO-seq with enhancers producing eRNAs rhythmically during the day (*p* value < 0.0001, *t* test). **d** Phase distribution of eRNAs produced from enhancers contacting all diurnal and nocturnal circadian promoters (left), circadian genes oscillating at the intronic level (centre) and circadian genes detected by GRO-seq (right) (all *p* values < 0.001, Wilcoxon ranked sum test). **e** Partial virtual 4C landscape of the *Rnf125* gene promoter at the four timepoints during the day. Acrophase is written next to the gene name. Significant contacts with enhancers producing oscillatory eRNA are shown. The majority of the eRNAs present a peak in transcription at ZT0 as *Rnf125* does. Genomic tracks show significant contacts as arcs and chromatin features including liver H3K4me3, H3K4me1, H3K27ac, DNaseI, eRNAs, and TADs. **f** Number of total significant interactions for core clock circadian gene promoters and a random control set of non-core-clock circadian gene promoters (*p* value < 0.0001, Mann-Whitney test). **g** Proportion of dynamic contacts for core clock genes and a random control set of non-core-clock circadian gene promoters (*p* value < 0.0001, Mann-Whitney test). **h**, **i** Virtual 4C for *Arntl and Nr1d1* core clock circadian gene promoters at all timepoints during the day. Features displayed are the same as described in **e**

Besides enhancers and open chromatin marks, we found that promoter-interacting regions were enriched for the occupancy of core clock TFs [13] (Fig. 3c, left panel, pval<8e−100, *t* test). When measuring the same enrichments at only circadian gene promoters interacting regions, both for the full set of our circadian genes and a subset corresponding to genes oscillating at the intronic level (see “Methods”), we found a significant preference for circadian gene promoters to contact with enhancers both detected by eRNA transcription or histone modifications, as well as regions occupied by core clock TF (Fig. 3b, c, right panels; all *p* values <8e−216, *t* test). We next assessed the rhythmicity of contacts between regulatory elements and circadian promoters during a circadian cycle. We identified dynamic genomic contacts involving circadian promoters using two distance regimes as described (see “Methods”). In total, we found 13,782 stable and 6047 dynamic contacts for 1195 circadian promoters and found enhancers preferentially engage in dynamic contacts (Fig. 3d). We next analyzed the number of interactions made by circadian promoters at different timepoints. We found that the number of contacts of circadian gene promoters peak during or around the phase of maximal mRNA abundance (chi square, *p* < 0.001) (Fig. 3e) (see different examples of circadian gene promoter expression profiles Additional file 3: Figure S3 and virtual 4C interaction landscapes across the paper Fig. 4e,h,i, Additional file 6: Figure S6F, Additional file 7: Figure S7A-F). This suggests that more regulatory elements are contacted at the time of peak transcription contributing to circadian gene regulation.

Next we identified transcription factor binding motifs at genomic regions involved in constant or rhythmic interactions with circadian promoters in an unbiased manner using the MEME suite (“Methods”) [30]. We found TF binding motifs both in regions forming constant and dynamic contacts with circadian promoters. Other DNA binding motifs were found enriched in regions engaged in either dynamic or constant contacts with circadian gene promoters (Fig. 3f, Table S5). The binding motif of the nuclear receptor Nr5a2/LRH-1 was highly enriched at interacting regions of rhythmic gene promoters. Nr5a2 is a key metabolic sensor that modulates bile acid synthesis and cholesterol homeostasis by regulating the expression of *Cyp7a1* and *Cyp8a1* circadian genes among others, and controls triglyceride synthesis and lipid composition and metabolism [31–34]. Interestingly, a pro-inflammatory function of Nr5a2 deficiency is linked to non-alcoholic fatty liver disease [35]. In addition, loss of Nr5a2 in the adult liver leads to disruption of hepatic lipid homeostasis and composition [36]. To confirm Nr5a2 occupancy at circadian gene promoters and interacting regions, we performed Chromatin IP (ChIP-qPCR) against Nr5a2 at the four timepoints during the day for three loci in which the DNA binding motif was found enriched. We found Nr5a2 dynamically occupying the *Arntl* circadian gene promoter with peak occupancy at ZT6 when *Arntl* gene expression starts to decrease (Fig. 3g, Additional file 3: Figure S3). A similar binding pattern over time was observed on the *Arntl* interacting enhancer (Fig. 3g). We then analyzed Nr5a2 binding to a chromatin region stably contacting the circadian gene promoter of *Ppp1r3c*. Interestingly, we found that Nr5a2 binding was dynamic with maximum occupancy of Nr5a2 at this region is also dynamic and peaks at ZT6 when *Ppp1r3c* gene expression starts to decrease (Fig. 3g, Additional file 3: Figure S3). In addition, we assessed Nr5a2 occupancy in a chromatin region dynamically interacting with the circadian gene promoter of *Gsk3a* but did not find the receptor bound to this region (Fig. 3g). Thus, our results suggest that Nr5a2 is dynamically occupying regulatory elements of circadian genes irrespective of whether the contacts to circadian gene promoters are constant or rhythmic over time.

Other binding sites for nuclear receptors enriched in regions contacting circadian promoters included VDR (vitamin D receptor) and ESR2 (estrogen receptor 2).

The Tcfap2c/AP-2 gamma binding motif was found to be highly enriched at dynamic interacting regions of circadian gene promoters. In the liver, Tcfap2c has been associated with repression of fatty acid synthesis pathways [37] and was identified as a key TF involved in lipid droplets biogenesis [38]. Fos:Jun/AP1 binding motifs were found in genomic regions forming stable contacts with circadian promoters. AP1 factors are a well-characterized immediate-early transcription factors induced in response to signals in the serum and that regulate the expression of circadian genes in liver and cultured cells [39] as well as the suprachiasmatic nucleus [40–42]. Recently, AP-1 was shown to bring together key genes and enhancers through stable and dynamic loops during macrophage development bringing together key macrophage genes and enhancers [43].

In summary, a set of DNA binding motifs for distinct liver nuclear receptors and immediate early genes are enriched in regions contacting circadian promoters and could function in the wiring of the circadian promoter 3D interactome in the liver.

### Circadian gene promoters interact with diurnal and nocturnal enhancers in the nuclear space

A set of enhancers are transcribed in a circadian fashion in the mouse liver [27]. We found that these enhancers preferentially contact circadian gene promoters, suggesting that rhythmically transcribed genomic regions, protein-coding and non-coding, interact with each other in the nuclear space (Fig. 4a). This is also true for our subset of circadian genes oscillating at the intronic level (see “Methods”) and for circadian genes detected through GRO-seq [27] reflecting primary transcriptional oscillation (Fig. 4b,c).

We then compared the transcriptional phases between promoters of circadian genes and their corresponding contacted enhancer elements with rhythmic transcription. To do so, we separated the circadian gene promoters into diurnal and nocturnal depending on their transcriptional acrophase and then analyzed the time of maximal RNA expression of their contacted enhancer elements. We found a significant contact preference between diurnal promoters and diurnal enhancers as well as nocturnal promoters and nocturnal enhancers (Fig. 4d, left). These preferences were more pronounced when analyzing our circadian intronic gene set reflecting primary transcription (see “Methods”) (Fig. 4d, center) or detected by GRO-seq (Fig. 4d, right, all *p* values < 0.0001 Wilcoxon signed rank test). For example, the *Rnf125* circadian gene promoter with peak transcription at ZT0 contacts 12 rhythmically expressed enhancers with acrophases between 19 and 1 h during the circadian cycle. Furthermore, as it can be observed, the number of contacts with the enhancers, increase during the acrophase (Fig. 4e).

### The core clock gene promoter contacts

Finally, we focused on the genomic interactions formed by circadian core clock gene promoters including *Npas2*, *Clock*, *Arntl*, *Cry1*, *Cry2*, *Per1*, *Per2*, *Rorc*, *Nr1d1*, *and Nr1d2* as defined by [44]. Notably, all core clock genes displayed fewer overall contacts compared to a random set of the same number of other circadian genes in the liver (12 vs 19.6 mean number of contacts for core-clock vs other circadian genes, Fig. 4f, *p* < 0.0001, *t* test). However, the contacts formed by the core clock gene promoters were more dynamic than a random set of the same number of contacts for other circadian genes in the liver (42.3% vs 26.8% mean proportion of dynamic contacts for core clock vs other circadian genes, Fig. 4g, *p* < 0.0001, *t* test). For instance, the *Arntl* circadian gene promoter engages in contacts with two enhancer elements at ZT18, the time when *Arntl* expression increases, in three contacts at ZT0, at time of maximal transcriptional output, and does not engage in contacts at either ZT6 or ZT12, when *Arntl1* transcription decreases (Fig. 4h, see Additional file 3: Figure S3 for the expression profile). The *Nr1d1* gene promoter engages in more contacts at the gene’s maximal time of expression, around ZT6 (Fig. 4i, see Additional file 3: Figure S3 for the expression profile). In contrast to core clock contact profiles (additional examples are shown for *Rorc, Nr1d2, Npas2,* and *Per2* (Additional file 7: Figure S7A-D, see Additional file 3: Figure S3 for expression profiles), promoters of circadian output genes engage in numerous contacts that are constant during the day as exemplified by *Dhr3* and *Ppp1r3c* gene promoters (Additional file 7: Figure S7E and F, see Additional file 3: Figure S3 for expression profiles). In conclusion, core-clock gene promoters engage in more dynamic genomic contacts compared to other circadian genes in the liver.

## Discussion

Circadian fluctuations in gene expression in the adult liver orchestrate essential physiological metabolic responses in the body. While molecular mechanisms underlying the circadian clock circuitry have been described at transcriptional and post-transcriptional levels, less is understood on how different genome structures contribute to or reflect cyclic gene expression [6]. Here we analyzed the genome conformation in mouse adult liver throughout a circadian cycle and reveal the properties of the circadian cis-regulatory chromatin landscape at different genomic scales associated with circadian rhythmicity in gene expression.

**Fig. 5:**
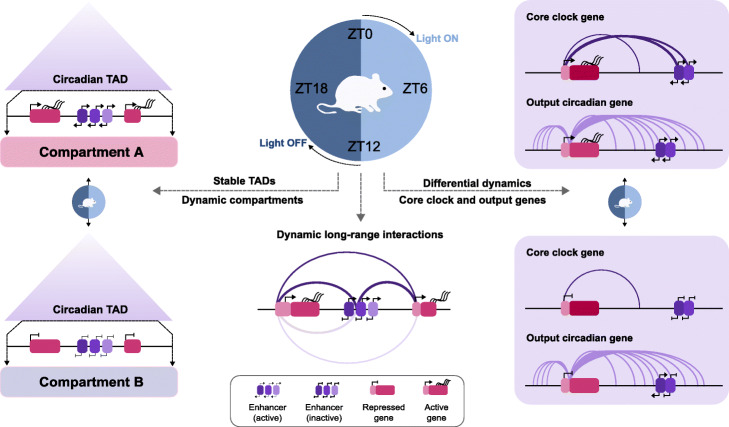
Chromatin conformation dynamics during a circadian cycle. Circadian TADs containing one or more circadian genes remain stable but overlap with regions that switch chromatin compartments in correlation with transcriptional activation and gain of open histone modifications marks during the 24 h (left). Inside cTADs, circadian genes and regulatory elements with similar acrophases contact each other in the nuclear space with interactions increasing at the time of peak transcription (middle). Core clock genes display highly dynamic contacts during the 24 h. In contrast, circadian output genes have more saturated contact profiles that remain stable during the day (right)

We found that 17% of the genome fluctuates between A and B compartments during a 24-h cycle. The genomic regions with changing compartment assignments throughout the day (OCCs) overlap with circadian TADs whose domain boundaries in contrast remain unchanged during a cycle. Switches between closed and open states of genomic compartments have been reported during organism development, cell differentiation, and cell cycle [11, 12, 45]. However, our results reveal that dynamic changes between compartment states occur also within hours and without cells dividing or changing their identity (Fig. 5).

Inside cTADs, circadian genes tend to be alone or sharing the TAD with other circadian genes and regulatory elements that are transcribed at similar times during the day, suggesting spatial isolation of temporal transcription control. The contacts formed by circadian gene promoters can remain constant or change over time (Fig. 5).

We performed an unbiased identification of TF binding motifs to discover candidate protein factors enriched at the anchor sites of chromatin loops involving circadian genes engaged in both dynamic and stable contacts. We found that the DNA binding motif of the metabolic liver nuclear receptors (Nr5a2/LRH-1) is highly enriched in both constant and dynamic circadian gene promoter interactions. We confirmed Nr5a2 occupancy at three loci in which the motif is present and found Nr5a2 dynamically occupies chromatin with maximum binding at ZT6 at the *Arntl* circadian gene promoter and dynamically contacted enhancer, as well as in a constant interaction from the *Ppp1r3c* circadian gene. These suggest a possible role of Nr5a2 in circadian loop formation and further experiments will be needed to fully dissect the potential function of Nr5a2 in rhythmic chromatin looping. Also we found the immediate early genes AP2 gamma and Fos:Jun are enriched in chromatin regions stably or dynamically contacting circadian gene promoters over time. This suggests that distinct nuclear receptors and immediate early transcription factors participate in shaping the circadian 3D cistrome.

Finally, by comparing the interaction profiles between core clock and output circadian genes, we discovered that core clock genes tend to contact fewer different genomic elements and that core clock interactomes are more dynamic compared to output circadian genes in the liver (Fig. 5). This is in line with recent candidate-scale 4C chromosome conformation capture experiments for two core clock and output genes [46]. These results suggest a robust regulation of core clock gene transcription by a few specific regulatory elements, which dynamically contact the core clock promoters. Alternatively, the control of core clock gene expression might rely primarily on their respective promoters and less on distal genomic elements than for output genes. On the contrary, output circadian engage in numerous and constantly maintained contacts, suggesting that more regulatory elements are required to control their expression in a pre-established genome architecture.

## Conclusions

Our results show that chromatin architecture dynamics during a circadian cycle is coordinated with circadian oscillations in transcription at different genomic scales. Furthermore, we provide a high-resolution atlas of the regulatory elements connected to circadian gene promoters resolved in time.

## Methods

### Mice and tissue isolation

C57BL/6 male mice were maintained in the Babraham Institute Animal Facility and all applied procedures were approved considering the animal welfare practices according to the Home Office in the UK. Then, 8-week-old male mice were maintained under a 12-h light:12-h dark cycle for 2 weeks and fed ad libitum. Livers were dissected from three biological replicate pools (Pool A, B and C) composed of 2 livers each at four timepoints ZT0, 6, 12, and 18, with ZT0 = lights on and ZT12 = lights off. The liver tissue was chopped into 5–6-mm^3^ pieces and directly fixed in 2% formaldehyde for 10 min.

### In nucleus Hi-C

In nucleus, Hi-C library generation was performed as previously described [21]. Briefly, fixed adult liver tissue from three biological replicates at ZT0 0, 6, 12, and 18 was sieved through a 70-μM cell strainer and dounce homogenized in 10 ml of ice-cold lysis buffer with a tight pestle for a total of 30 strokes on ice. Nuclei were washed and permeabilized with 0.3% SDS for 45 min at 37 °C and then incubated overnight with HindIII at 37 °C, DNA ends were labeled with biotin-14-dATP (Life Technologies) in a Klenow end-filling reaction and ligated in nuclei overnight. DNA was purified by phenol-chloroform, and the concentration was measured using Quant-iT PicoGreen (Life Technologies). A total of 10 μg of DNA was sheared to an average size of 400 bp using a Covaris machine and following the manufacturer’s instructions. The sheared DNA was end-repaired, adenine tailed, and subject to a double size selection using AMPure XP beads to isolate DNA ranging from 250 to 550 bp in size. Ligated fragments marked by biotin-14-dATP were pulled-down using MyOne Streptavidin C1 DynaBeads (Invitrogen) and ligated to paired-end adaptors (Illumina). The in nucleus Hi-C libraries were amplified using the PE PCR 1.0 and PE PCR 2.0 primers (Illumina) using 6–9 PCR amplification cycles as required.

### Promoter Capture in nucleus Hi-C (Chi-C)

Promoter Capture was performed as previously described [21–23]. Briefly, Biotinylated 120-mer RNA baits were designed to target both ends of HindIII restriction fragments overlapping the Ensembl promoters of protein-coding and noncoding transcripts and UCEs as described in detail in [22]. Promoter Capture was carried out using in nucleus Hi-C libraries derived from three biological replicates at ZT0 0, 6, 12, and 18 with the SureSelect target enrichment system and the biotinylated RNA bait library according to the manufacturer’s instructions (Agilent Technologies). After library enrichment, a post-capture PCR amplification step was carried out using the PE PCR 1.0 and PE PCR 2.0 primers (Illumina) with 4–6 PCR amplification cycles as required. In nucleus Hi-C and CHi-C libraries were sequenced on the Illumina HiSeq 2000 platform.

### ChIP-seq

For ChIP-seq, liver tissue for two biological replicates at ZT0 and ZT12 was dissected as processed as for Hi-C and then fixed in 1% formaldehyde for 5 min. Chromatin immunoprecipitation was performed as described [21] using 10 μg of α-CTCF (Millipore, 07-729). DNA was purified using Zymo Research DNA purification columns. Sequencing libraries were prepared with the NEBNext ChIP-seq library prep kit (NEB) according to the manufacturer’s instructions. DNA was purified using AMPure beads (Agencourt). For quantitative chromatin immunoprecipitation experiments (ChIP-qPCR), liver tissue, collected at ZT0, ZT6, ZT12, and ZT18 per duplicate, was fixed with a 1% formaldehyde for 10 min. Cell lysis and chromatin immunoprecipitation were performed essentially the same way as for the ChIP-seq experiments, using 5 μg of a monoclonal antibody against the NR5A2 receptor (Santa Cruz Biotechnology, sc-393369) per assay. ChIP DNA templates were purified using the Zymo Research ChIP DNA Clean & Concentrator kit (Zymo Research, D5201). Quantitative protein occupancy at specific regions was performed by qPCR (Jena Bioscience, PCR-369), according to the manufacturer’s instructions. Mean values for all the analyzed regions across ZTs were expressed as fold enrichment compared to a mock control. Statistically significant differences between ZTs and mock were analyzed using one-way ANOVA followed by the Tukey post hoc test.

### RNA-seq

For RNA-seq libraries, total RNA was purified from the same livers processed for in nucleus Hi-C from four biological replicates at ZT0, 6, 12, and 18. Sequencing libraries were prepared with TruSeq Stranded Total RNA Ribo Zero Gold Library Prep Kit v2 (Illumina).

### Data processing

#### Hi-C analysis

Hi-C sequenced reads were mapped to the mouse genome (mm9) using HiCUP [47] with default parameters. Downstream processing was done using Juicer [48] and data was visualized using Juicebox [49]. Hi-C heatmaps at different bin resolutions were created and normalized using Knight-Ruiz (KR) matrix balancing algorithm from Juicer. Statistics for each library can be found in Table S1.

##### Metaplots

The metaplots were created using python custom scripts. Briefly, the script takes a feature of interest and calculates the frequency of interactions around it using as input the KR normalized Obs/Exp Hi-C matrices at different resolutions (10, 25, or 50 kb) from different timepoints (ZT0,6,12,18). The final metaplot is the median value of all the plots for the list of anchors. For the TAD-anchored metaplots, Hi-C normalized matrices at 50 kb resolution were used. Each TAD (see TAD calling) was scaled to fit into 5 bins, and only 1000 TADs from all datasets and using all chromosomes were randomly chosen to reduce computing time. For the CTCF- anchored metaplots, Hi-C normalized matrices at 10 kb resolution were used. Each CTCF peak (see ChIP-Seq analysis) was scaled to fit into a single bin, and only 1000 CTCF peaks identified at ZT0 and ZT12 were randomly chosen to reduce computing time. The matrices generated were plotted using heatmap.2 from the package plots.

#### TAD calling

For all timepoints, we retrieved Knight-Ruiz normalized contact matrices from Juicer for all chromosomes at 25 kb and 50 kb resolution. TADs were identified using TADtool [50] with the insulation score algorithm. To find appropriate parameters for TAD identification, we called TADs for chromosome 1 across all timepoints using contact matrices at 25 kb and 50 kb resolution and a window size of 100, 150, 155, 175, 195, and 200 kb over threshold values from 70 to 200. For all data sets at 50 kb resolution, we called TADs with a window size value of 200 kb and a threshold value of 140 while for all data sets at 25 kb resolution, we called TADs with a window size value of 100 kb and a threshold value of 76. We found that these parameters show good agreement between identified TADs and visual inspection of Hi-C datasets in Juicer. Of note, visual inspection of Hi-C datasets with TADs identified at 25 kb resolution reveals that these represent sub-TADs contained within TADs identified at 50 kb resolution.

#### TAD analysis

##### Size distribution

The number of TADs per timepoint and the size distribution of TADs across a circadian cycle was calculated using a custom R script using a Wilcoxon rank sum test.

##### Circadian TADs

Circadian TADs were defined as previously described [9]. TADs containing at least one circadian gene as identified by our RNA-seq analysis were classified as Circadian TADs. Each Circadian TAD was further categorized depending on the number of circadian genes contained within the TAD. Then we considered the transcriptional phases of the circadian genes within Circadian TADs and classified them as same or different. The probability to have the same or different transcriptional phases within cTADs was calculated considering the total number of circadian genes per phase. The observed over expected significance was estimated performing a chi-square test.

##### Overlap

To determine the number of unique and shared TADs between the timepoints, we calculated the overlap in different pair-wise comparisons using the Venn module of Intervene [51]. A TAD was considered shared between timepoints if more than 80% of the genomic domain region overlapped with a domain from a different Hi-C data set.

#### Compartment analysis

Compartments were identified applying PCA to the normalized interaction matrices at a 100 kb resolution using Juicer [48]. PCA1 was used to assign A and B compartments. To verify the reproducibility of the compartment call, the PCA analysis was applied on the separate replicates and just the merged data was used for downstream analysis. A custom script and publicly available ChIP-seq BAM files for H3K4me3 [13] were used to set the sign to the compartments identified by Juicer. A total of ~ 20,000 compartments were identified at each timepoint. We identified significantly changing compartments as those genomic regions with a change in PCA1 across different timepoints consistently in the three biological replicates through a one-way ANOVA test.

##### Transcription in A and B compartments

To relate compartments A and B with transcription, we calculated the log2 RPM (reads per million) values for all regions assigned to compartments A and B per timepoint (ZT0,6,12,18) using SeqMonk (https://www.bioinformatics.babraham.ac.uk/projects/seqmonk/) and RNA-seq BAM files (see “RNA-seq data processing”) per timepoint as input and to applied a Kruskal Wallis test for all compartments and a Mann-Whitney test for OCCs. The distribution of log2 RPM values per compartment type at each timepoint is presented as violin plots.

##### Correlation with histone marks

To relate changes in compartment status with the enrichment of histone post-translational modifications, we calculated the RPM (reads per million) values for all regions assigned to compartment A and B per timepoint (ZT0,6,12,18) using SeqMonk and publicly available ChIP-seq datasets for the histone post-translational modifications H3K4me3 and H3K4me1 [28] per timepoint as input and applied a one-way ANOVA test and a Tukey post hoc test.

##### Correlation with HDAC3

To relate changes in compartment status with the enrichment of HDAC3, we calculated the log2 RPM (reads per million) values for all regions assigned to Compartment A at ZT0 and that change to Compartment B at Z12 using SeqMonk and publicly available ChIP-seq datasets for the histone deacetylase HDAC3 [52] and applied a Wilcoxon test.

#### Promoter CHi-C

The sequenced reads were processed using HiCUP [47]. The filtering and identification of significant interactions were performed with CHiCAGO [24]. To identify differential interactions, the script implemented by [21] was used. This script can identify the differential interactions from a Promoter Capture Hi-C dataset, using the edgeR package [53] to statistically quantify changes in reads for the interactions. To increase the confidence in dynamic interactions, we filtered the dataset only including baits overlapping circadian genes. To account for the distance bias in the read count, we also divided the CHi-C interactions into greater or less than 150 kb groups. These preliminary results were filtered by FDR and fold change; both distance regimes were combined. To plot long-range interactions, we used the Washington Epigenome Browser (http://epigenomegateway.wustl.edu/browser/) using the mouse genome version mm9 and as input properly formatted CHiCAGO output files.

##### Characterization of interacting regions

To characterize the type of genomic element that promoters contact derived from our Promoter CHi-C, we calculated the observed/expected number of overlaps between the other ends (the genomic segment interacting with a promoter) and a set of genomic regions occupied by Transcription Factors or enriched for histone post-translational modifications using a custom python script. The expected number of overlaps is calculated by generating a random distribution of other end CHi-C fragments considering two conditions: (1) the length of the other end fragment observed in the original dataset; (2) the distance between the baits (promoter) and other ends. With this random set, we repeat the overlap with the features of interest and keep the number of “expected” overlaps by chance and repeat the process at least 100 times. To plot the results, the mean was calculated for all the iterations of the expected values. The significant differences were calculated using a *t*-test between the distribution of the expected values and the observed number of overlaps.

##### Interactions per promoter

To calculate the number of interactions per promoter at each timepoint, python custom scripts were used. Circadian promoters were defined as genes with circadian transcription as identified by our RNA-seq analysis. For core clock promoter interaction analysis, we used the classic core clock list described in [44]. A list of enhancers with circadian transcription (eRNAs) was used derived from [27]. The comparison with non-core-clock-circadian genes includes all the other circadian genes determined from our RNA-seq analysis. To calculate the distribution of the expected number of interactions made by non-core-clock-circadian genes, we randomly selected 11 genes from the entire set; this procedure was repeated 100 times. The comparison between the observed number of interactions made by the core clock genes and the distribution of expected interactions of non-core-clock-circadian genes was made using a Mann-Whitney test.

##### Analysis of interactions between circadian promoters and enhancers producing eRNAs

The enhancer regions with transcribed eRNAs from [27] were assigned to the other ends (interacting region) and then the bait (promoter) from our Promoter CHi-C datasets. To calculate the observed/expected number of interactions between promoters and enhancers with oscillatory eRNAs (osceRNAs) and non-oscillatory eRNAs (nonosceRNAs) that map to the CHi-C dataset, the expected number was calculated by taking the same number of osceRNAs from the nonosceRNA set and count the number of interactions between the restriction fragment containing an enhancer transcribed into eRNAs and the fragment containing circadian promoters derived from the CHi-C dataset. This process is repeated at least 100 times. Each enhancer region with transcribed eRNAs was assigned with a phase (maximal transcription during a circadian cycle) [27] as well as the promoter of the CHi-C datasets using our RNA-seq analysis. To make eRNA phases [27] more comparable to the circadian promoters identified by our RNA-seq analysis, we grouped them into eight groups each containing three timepoints. First, we mapped the osceRNAs to the other ends of the CHi-C, then retrieved the bait fragment associated with that eRNA and filtered the fragments overlapping with the set of circadian promoters identified by our RNA-seq analysis. Then, we divided the elements in diurnal and nocturnal to have a better understanding of the interaction profiles and perform a Wilcoxon rank sum test per pair of elements for each category.

#### Virtual 4C

The output BAM files from HiCUP for the different promoter CHi-C libraries per timepoint were used as input for SeqMonk to create Virtual-4C plots using promoters of interest as viewpoints to display raw promoter Chi-C counts as for the Arntl1 virtual 4C (Additional file 4: Figure S4A). Alternatively, the view point of interest and its significant interaction partners were filtered from the CHiCAGO output file and visualized in the Washington Epigenome Browser (http://epigenomegateway.wustl.edu/browser/) using the mouse genome version mm9.

#### Construction of promoter-promoter interaction networks

The graphs were constructed using the output generated by CHiCAGO. The raw interaction files for each timepoint were processed to adjust gene names using ad hoc Python scripts due to many transcript variants presented in those files. Also, a nomenclature was established to represent ultraconserved elements (UCEs). Each UCE was represented according to its locus using the following format: uce_[Chromosome number_[Position of first nucleotide]. Each undirected graph was constructed, analyzed, and visualized using the NetworkX Python module [54]. The graphs generated were disconnected and contained a large number of small components. For this reason, the components containing 3 or fewer nodes were filtered out for presentation purposes. The statistical analysis used to identify differences in node properties between timepoints was carried out using SciPy [55] and StatsModels modules [56]. Finally, the ontology enrichment analysis was conducted with the g:Profiler web tool [25] using the POST request API against the *Mus musculus* genome (*mmusculu*s) with other parameters kept as default. To evaluate the phase coherence between circadian gene promoters contacting each other, we assigned the phases of circadian genes with our RNAseq and plotted the phase distribution of circadian promoters contacted by either diurnal or nocturnal circadian promoters. We applied a Wilcoxon rank sum test to calculate the difference between pairs of the two categories.

#### ChIP-seq data analysis

Raw sequencing data files for all samples were first processed with FastQC for general quality controls. Sequencing reads were mapped against the mouse genome (NCBIM37/mm9) using Bowtie2 [57] with default parameters for single and paired reads. Mapped reads were filtered by map quality (-q 30) using samtools (samtools view). Bam files were sorted (samtools sort) and indexed (samtools index). Duplicates were removed with Pickard. Bam files were imported to deepTools v3.3.1 [58] to create signal tracks with bamCoverage. Signal tracks for all data were visualized using IGV [59]. Peak calling was performed using MACS2 callpeak function with default parameters [60]. Peak overlap analysis was performed using the Venn module of Intervene (Khan & Mathelier, 2017). The MEME-ChIP tool [61] was used for motif analysis using the fasta sequences from peaks detected by MACS2 with default parameters.

#### RNA-seq data processing

Raw sequencing data files for all samples were first processed with FastQC for general quality controls. Sequencing reads were mapped against the mouse genome (NCBIM37/mm9) using TopHat [62] with default parameters. Deeptools [58] and BAM files for all samples were used to calculate Spearman’s correlation between all biological replicates for each timepoint. All samples were highly correlated (Spearman’s correlation > 0.85). Heatmaps of correlations were created using the Deeptools plotCorrelation. To create bigWig signal tracks for all timepoints, we merged all biological replicates per timepoint using samtools [63]. Merged bam files per timepoint were processed with Deeptools bamCoverage to create strand-specific and RPKM normalized signal tracks suited for comparison. Visualization of signal tracks was done using IGV genome browser [59]. Mapped reads for all samples were then used to assemble and quantify expressed genes and transcripts using StringTie [64] with default parameters. Differential expression was performed with Ballgown [65] in R using StringTie table counts for all samples. Genes with differential expression between at least one pair of timepoints were identified after correction for multiple hypothesis testing with a *q*-value < 0.01. We classified a gene as circadian if it was differentially expressed between at least a pair of timepoints. We assigned a phase for each differentially expressed gene to the timepoint with the highest average FPKM value. Heatmap of circadian genes was created using pheatmap function in R using as input a list of 1256 differentially expressed genes at a *q*-value < 0.01 and ordered by phase. Expression values for all genes were *Z*-score corrected. Plots of FPKM expression over time for selected examples were generated using library ggplot2 in R. To generate the subset of circadian genes oscillating at the intronic level, we filtered out our list to obtain genes matching the intronic circadian gene set reported in Koike et al. [13].

#### Gene Ontology analysis

Gene ontology enrichment analysis and pathway enrichment were done using DAVID [66]. All significant biological processes and pathways had a *p* value < 0.01. Barplots were generated with ggplot2 in R.

#### Motif analysis

The fasta sequence files from the HindIII fragments of the *otherend* of stable, dynamic, and both types of chromatin contacts were downloaded using the mouse genome version mm9. The MEME-ChIP tool [61] was used for motif analysis with default parameters.

## Supplementary Information

**Additional file 1: Figure S1.** OCCs present fluctuations in histone modifications. A. Heatmap of PC1 values for stable compartments from the three independent Hi-C biological replicates and B, the merged Hi-C biological replicates. C. PC1 eigenvector correlations between every pair of timepoints (Kruskal-Wallis rank sum test p-value < 2e-16). D. Proportion of OCCs and constant compartments in the mouse genome. E. Barplot of the frequency of the different OCCs categories with compartments switching at different times of the 24 hours. For example AABA refers to OCCs with compartment assignment A at ZT0, A at ZT6, B at ZT12 and A at ZT18. F. Chromatin features (H3Kme4 and H3Kme1) at OCCs across time points (AABB, ABBB and ABAB OCCs are shown) (*** all p-values < 0.001 except for the H3K4me1 AABB, one way ANOVA and Tukey post hoc test).

**Additional file 2: Figure S2.** Total RNA-seq analysis at four timepoints during the circadian cycle. A. Spearman correlation analysis of the four biological replicates from ZT0 and ZT12 (r ≥ 0.85). B. Heat map of the relative transcription (Z scores) of 1,257 oscillating genes sorted by oscillation phase. C. Individual expression profiles and genome browser tracks showing examples of the RNA-seq signal for *Arntl, Per1, Nr1d1, Cry1, Ppp1r3c* and *Gsk3a* oscillating genes (Fragments Per Kilobase of transcript per Million mapped reads, FPKM) (n=4, q-value <0.01). D. GO analysis for the detected oscillating genes. Significantly enriched categories are shown. E. KEGG analysis for detected oscillating genes. Significantly enriched categories are shown. F. Additional individual transcriptional profiles from the RNA-seq for *Rorc*, *Clock*, *Npas2*, *Cry2*, *Per2*, *Per3* circadian genes. G. RT-qPCR validation for candidate circadian genes both at mRNA and pre-mRNA level (p-values < 0.0001, one-way ANOVA test).

**Additional file 3: Figure S3.** Expression profiles of the circadian genes displayed along the paper. RNA-seq signal tracks for circadian genes shown across the paper for the four timepoints during the circadian cycle. *Arntl*, *Npas2*, *Cgn*, *Nr1d1*, *Dhrs3*, *Nr1d2*, *Per2*, *Ppp1r3c*, *Tef*, *Rnf125*, *Rorc* and *Aco2* circadian gene expression profiles are presented.

**Additional file 4: Figure S4.** TAD structure and CTCF binding is preserved during the 24 hours. A. TADs overlap between time points. B. TADs size distribution at all time points. C. 50kb resolution Median Observed/Expected Hi-C signal around 1000 randomly selected TADs from ZT6, 12 and 18 plotted on the indicated time points. TADs were scaled to fit the five central bins. D. CTCF ChIP-seq motif analysis and peak overlap between ZT0 and 12. Genomic tracks of CTCF ChIP-seq signal at example regions harbouring circadian genes. E. Above, the same metaplots as in C but for 1000 randomly CTCF peaks found at ZT12 plotted using ZT12 and ZT0 Hi-C contacts. Below, metaplot using CTCF peaks from mESC and plotted using liver ZT0 and 12 Hi-C contacts. CTCF peaks are at the central bin of the metaplot. F. TAD and cTAD size comparison (p-value < 2.2e-16, Wilcoxon rank sum test).

**Additional file 5: Figure S5.** Promoter-Promoter networks in the mouse liver over a circadian cycle. A. Partial view of a virtual 4C from the *Arntl* gene promoter using the Hi-C and P-CHi-C raw valid pairs. Histograms of read counts per restriction fragment around the bait region corresponding to the captured promoter are shown. B. Number of total read counts comparing Hi-C and P-CHi-C recovered using the *Arntl1* gene promoter as bait on a virtual 4C (restriction fragments with at least 5 reads in the P-CHi-C experiment where used) (p-value < 0.0001, Wilcoxon ranked test). C. Promoter-promoter contact network at ZT0. Each color represents a chromosome. Nodes with degree=1 are not included for simplicity. D. The four largest promoter-promoter interaction clusters of the network at the different time points during the day. E. Significantly enriched GO categories for the genes on the most prominent promoter-promoter clusters shown in D.

**Additional file 6: Figure S6.** Circadian gene promoter-promoter interactions. A. Promoter-promoter contact network at ZT0 with the circadian genes marked in blue. B. Above, number of edges (interactions) between circadian gene promoters at ZT0, 6, 12 and 18 hours of the day (blue line) compared to a random set of non circadian promoters. Below, z-score for circadian gene promoter contacts compared to the random sampling. C. Quantification of the read counts supporting all circadian gene promoter-promoter interactions compared to a random set of non circadian promoter-promoter interactions (*** p-value < 0.001, Mann Whitney test) D. Transcriptional phase distributions of circadian promoters in contact with diurnal or nocturnal circadian promoters (all p-values < 0.0001, Wilcoxon signed rank test) for our circadian intronic gene set. E. The same as in D for the circadian gene set detected through GRO-seq (Fang et al., 2014) (all p-values < 0.0001, Wilcoxon signed rank test) F. Virtual 4C landscape for the *Tef* circadian gene promoter from P-CHi-C data at all time points during the day. The acrophase of *Tef* is written next to the gene name. Expression profiles for both genes can be found in Figure S3. Genomic tracks show significant contacts as arcs and chromatin features including liver H3K4me3, H4K4me1, H3K27ac, DNAseI, eRNAs and TADs. *Tef* gene promoter contacts *Aco2* gene promoter both with acrophase at ZT12.

**Additional file 7: Figure S7.** Core clock gene promoter vs output circadian genes interaction landscapes.A-D. Virtual 4C for *Rorc*, *Npas2, Nr1d2* and *Per2*, core clock circadian gene promoters from P-CHi-C data at all time points during the day. The *Rorc* circadian gene promoter contacts *Cgn* circadian gene promoter and both peak in transcription at ZT18. E-F Virtual 4C for *Dhrs3* and *Ppp1r3c* output circadian genes in the liver. Acrophases are written next to the gene names. Genomic tracks show significant contacts as arcs and chromatin features including liver H3K4me3, H3K4me1, H3K27ac, DNAseI, eRNAs and TADs. Expression profiles for all genes can be found in Figure S3. The interaction profiles of core clock genes display less contacts that dynamically change over time. The two output gene contact profiles show more saturated contacts that are mostly constant during the 24 hours.

**Additional file 8.** Custom code. This file contains a summary of the custom code used in this work.

**Additional file 9.** Review history.

**Additional file 10: Table S1.** Hi-C statistics. HiCUP summary results for the independent Hi-C replicates.

**Additional file 11: Table S2.** Total stranded RNAseq number of unique read pairs.

**Additional file 12: Table S3.** Circadian genes detected from total RNAseq.

**Additional file 13: Table S4.** Promoter Capture Hi-C statistics. HiCUP summary results for independent P- CHi-C replicates and significant interactions detected with CHiCAGO.

**Additional file 14: Table S5.** Transcription factor DNA binding motif enrichment analysis. This table contains the information from all time points for both dynamic and stable interactions separately.

## Data Availability

Further information and requests for resources and reagents should be directed and will be fulfilled by the lead contact Mayra Furlan-Magaril. Accession for all data sets presented in Mayra Furlan-Magaril, Masami Ando-Kuri, Rodrigo G. Arzate-Mejía, Jörg Morf, Jonathan Cairns, Abraham Roman-Figueroa, Luis Tenorio-Hernandez, A. César Poot-Hernández, Simon Andrews, Csilla Várnai, Boo Virk, Steven W. Wingett, and Peter Fraser. The global and promoter-centric 3D genome organization temporally resolved during a circadian cycle, including Hi-C, Promoter CHi-C, CTCF ChIP-seq, and RNA-seq are deposited Gene Expression Omnibus (GEO) with accession number GSE155161 [67]. All custom scripts and inputs used in Mayra Furlan-Magaril, Masami Ando-Kuri, Rodrigo G. Arzate-Mejía, Jörg Morf, Jonathan Cairns, Abraham Roman-Figueroa, Luis Tenorio-Hernandez, A. César Poot-Hernández, Simon Andrews, Csilla Várnai, Boo Virk, Steven W. Wingett, and Peter Fraser. The global and promoter-centric 3D genome organization temporally resolved during a circadian cycle are available at Github repository https://github.com/mandok/circadian_3Dchrom [68]. Also a summary of the custom code is included as an additional file (Additional file 8: Custom code).
