# Supplementary figures and images for: The global and promoter-centric 3D genome organization temporally resolved during a circadian cycle
Source: Genome Biol. 2021 Jun 8;22:162. doi: 10.1186/s13059-021-02374-3 (PMC8185950; doi:10.1186/s13059-021-02374-3)

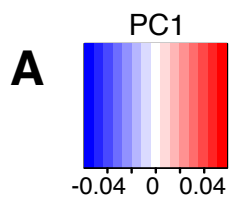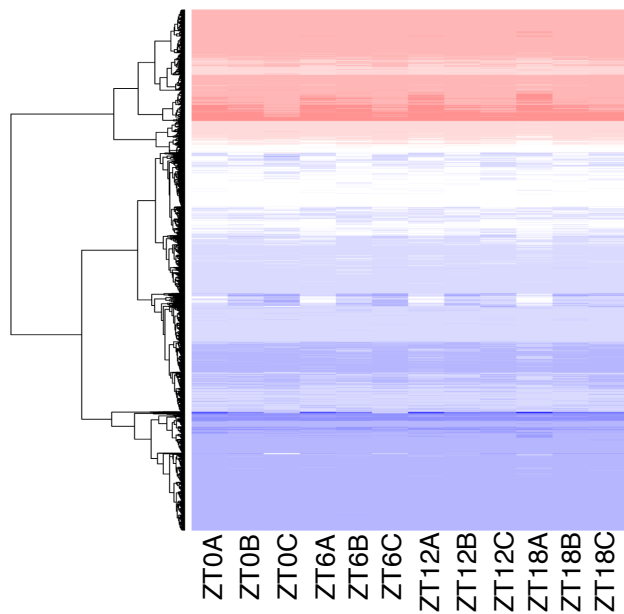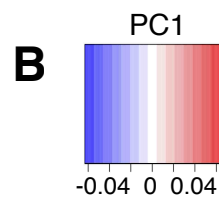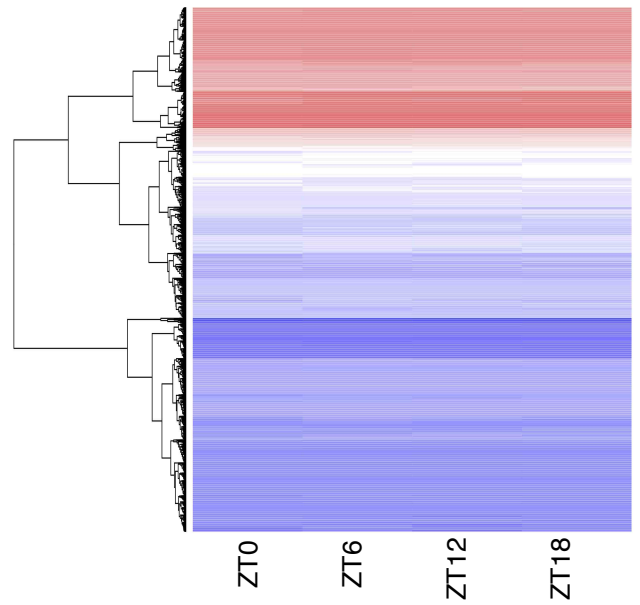

**C**

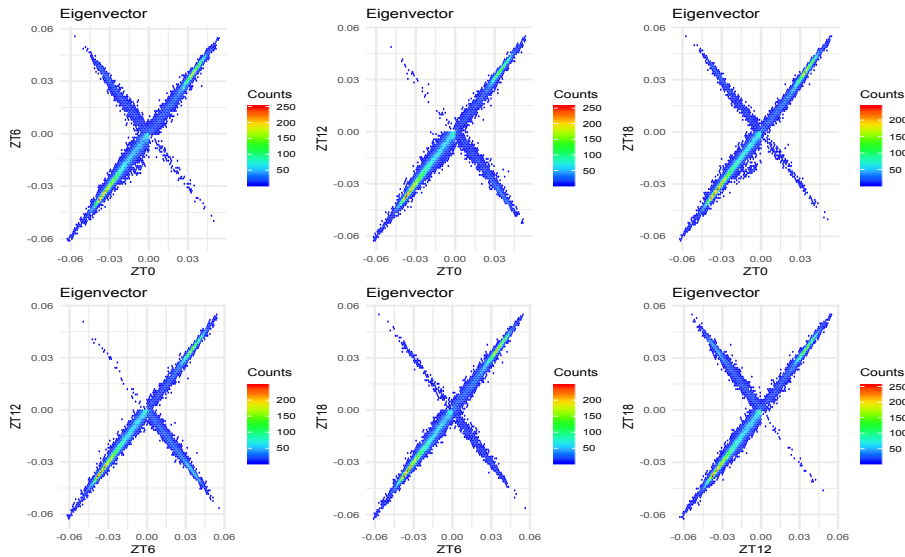

**D**

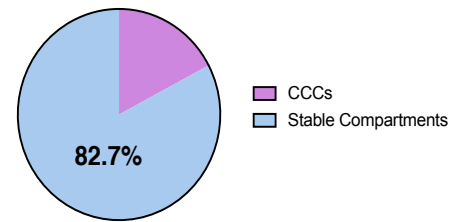

**E**

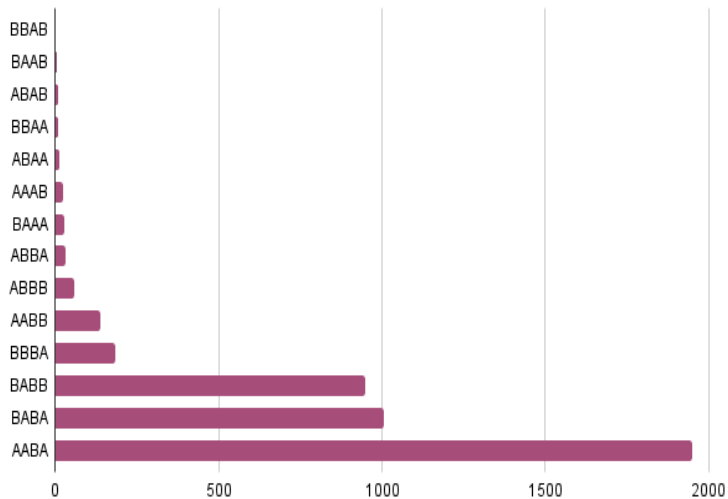

**F**

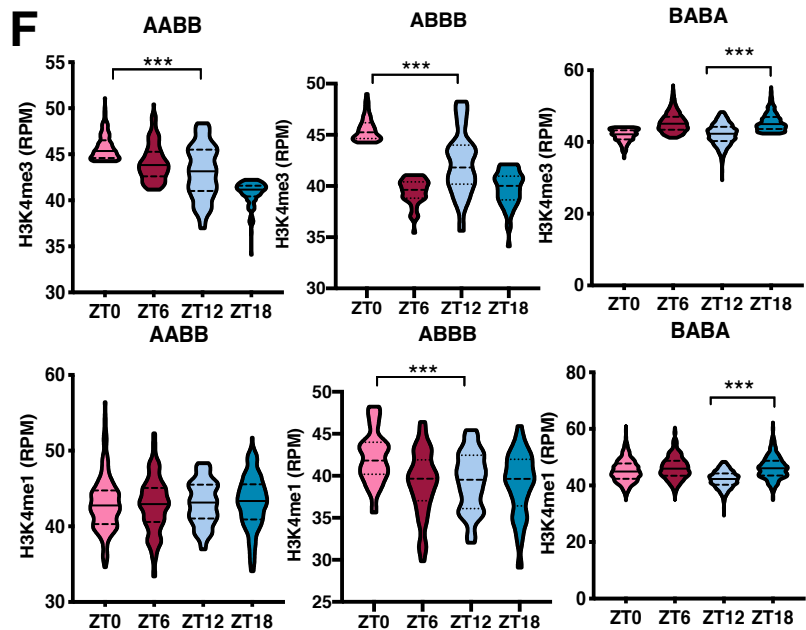

Supplement: Supplementary file 1 — Additional file 1: Figure S1. OCCs present fluctuations in histone modifications. A. Heatmap of PC1 values for stable compartments from the three independent Hi-C biological replicates and B, the merged Hi-C biological replicates. C. PC1 eigenvector correlations between every pair of timepoints (Kruskal-Wallis rank sum test p-value < 2e-16). D. Proportion of OCCs and constant compartments in the mouse genome. E. Barplot of the frequency of the different OCCs categories with compartments switching at different times of the 24 hours. For example AABA refers to OCCs with compartment assignment A at ZT0, A at ZT6, B at ZT12 and A at ZT18. F. Chromatin features (H3Kme4 and H3Kme1) at OCCs across time points (AABB, ABBB and ABAB OCCs are shown) (*** all p-values < 0.001 except for the H3K4me1 AABB, one way ANOVA and Tukey post hoc test). [file 13059_2021_2374_MOESM1_ESM.pdf]

**A**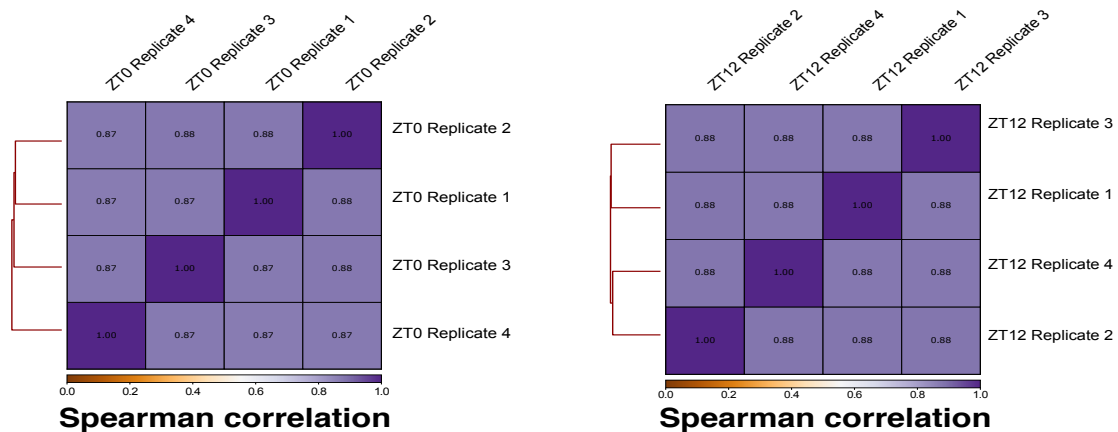**B**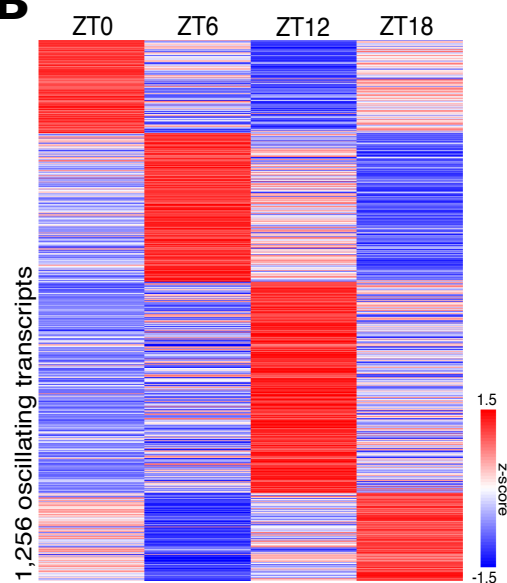**C**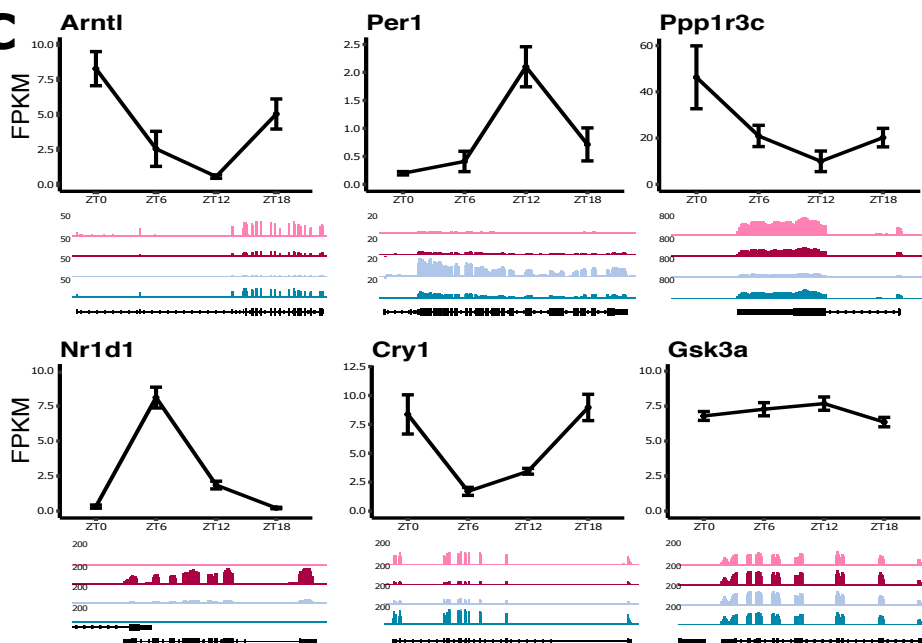**D**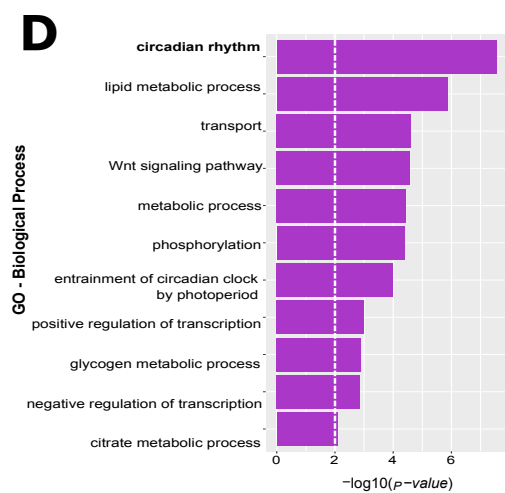**F**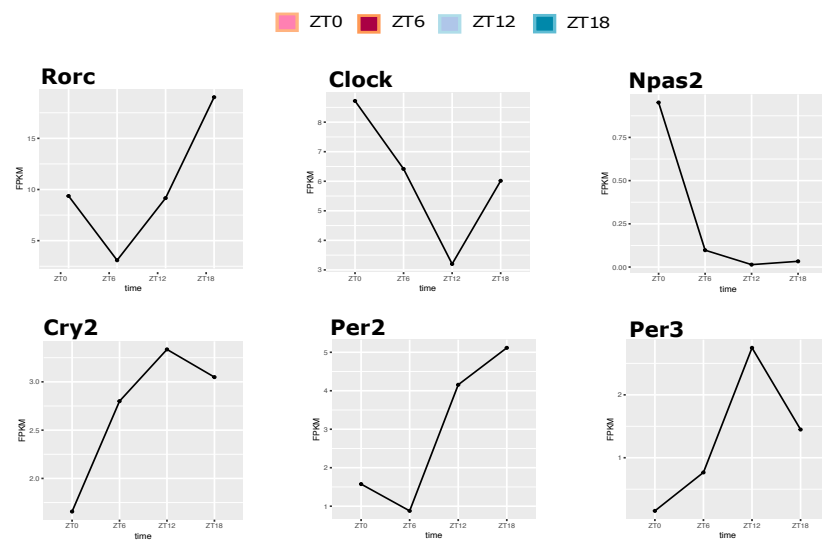**E**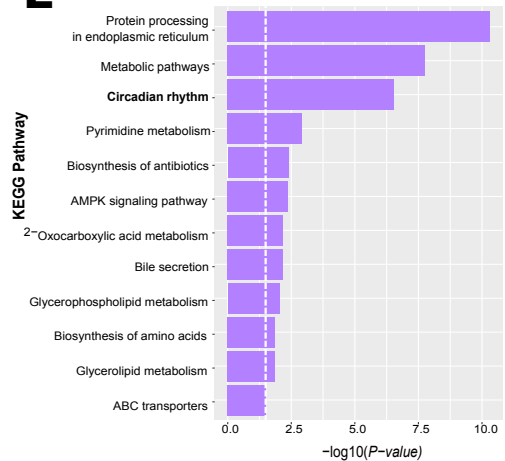**G**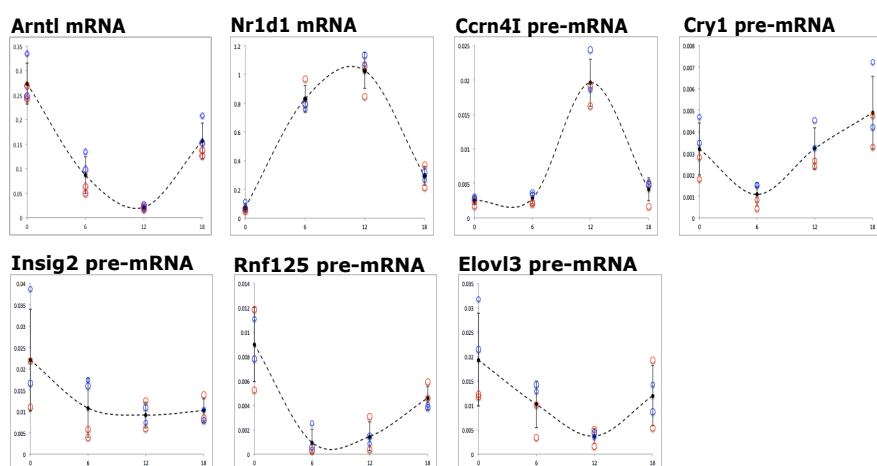

Supplement: Supplementary file 2 — Additional file 2: Figure S2. Total RNA-seq analysis at four timepoints during the circadian cycle. A. Spearman correlation analysis of the four biological replicates from ZT0 and ZT12 (r ≥ 0.85). B. Heat map of the relative transcription (Z scores) of 1,257 oscillating genes sorted by oscillation phase. C. Individual expression profiles and genome browser tracks showing examples of the RNA-seq signal for Arntl, Per1, Nr1d1, Cry1, Ppp1r3c and Gsk3a oscillating genes (Fragments Per Kilobase of transcript per Million mapped reads, FPKM) (n=4, q-value <0.01). D. GO analysis for the detected oscillating genes. Significantly enriched categories are shown. E. KEGG analysis for detected oscillating genes. Significantly enriched categories are shown. F. Additional individual transcriptional profiles from the RNA-seq for Rorc, Clock, Npas2, Cry2, Per2, Per3 circadian genes. G. RT-qPCR validation for candidate circadian genes both at mRNA and pre-mRNA level (p-values < 0.0001, one-way ANOVA test). [file 13059_2021_2374_MOESM2_ESM.pdf]

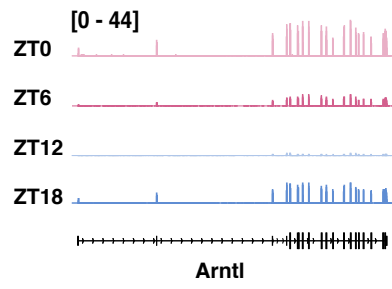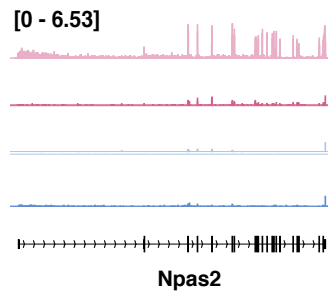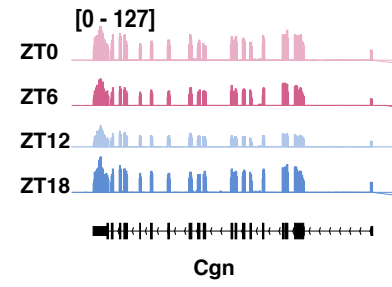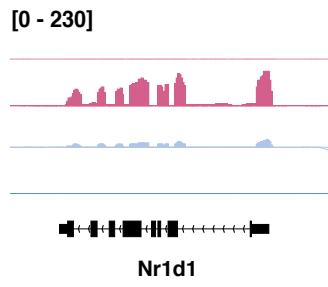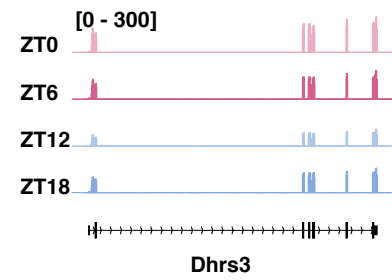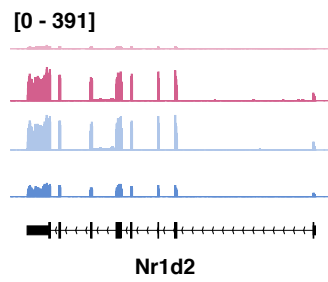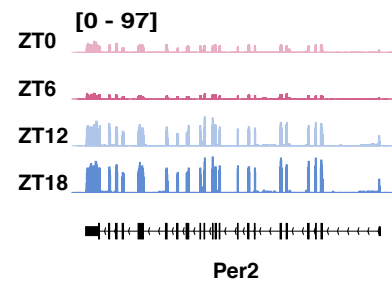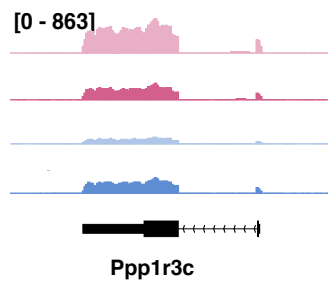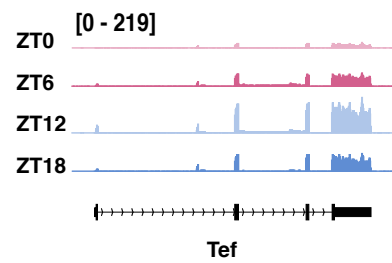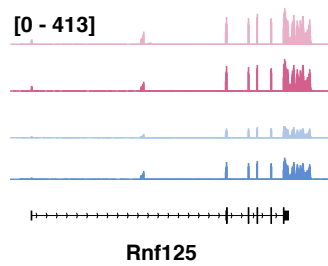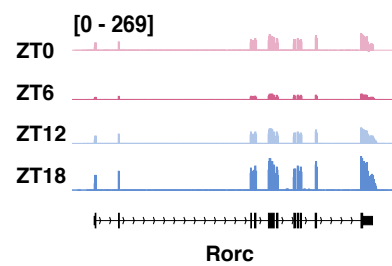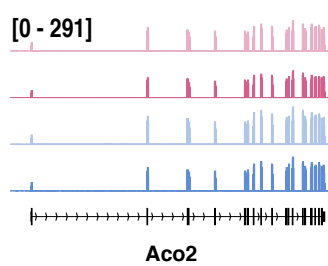

Supplement: Supplementary file 3 — Additional file 3: Figure S3. Expression profiles of the circadian genes displayed along the paper. RNA-seq signal tracks for circadian genes shown across the paper for the four timepoints during the circadian cycle. Arntl, Npas2, Cgn, Nr1d1, Dhrs3, Nr1d2, Per2, Ppp1r3c, Tef, Rnf125, Rorc and Aco2 circadian gene expression profiles are presented. [file 13059_2021_2374_MOESM3_ESM.pdf]

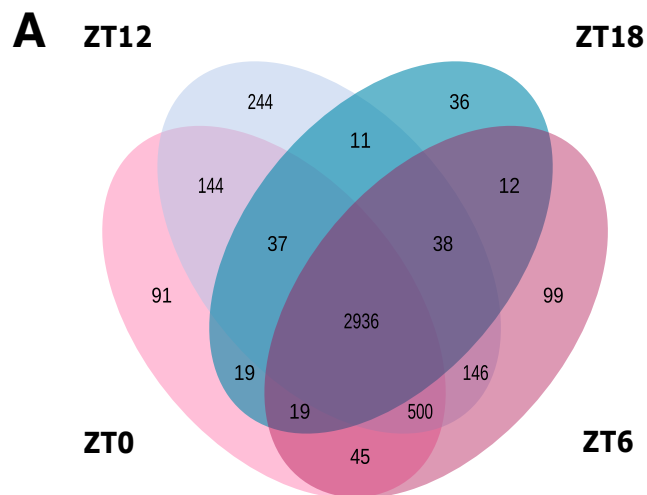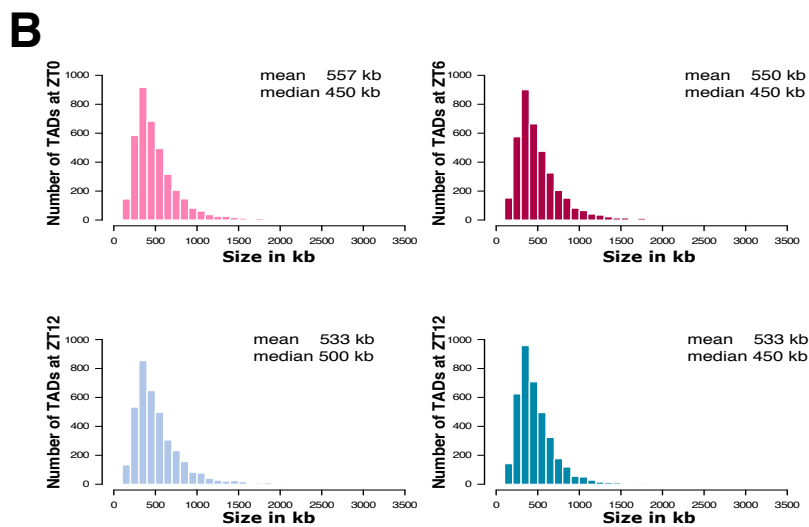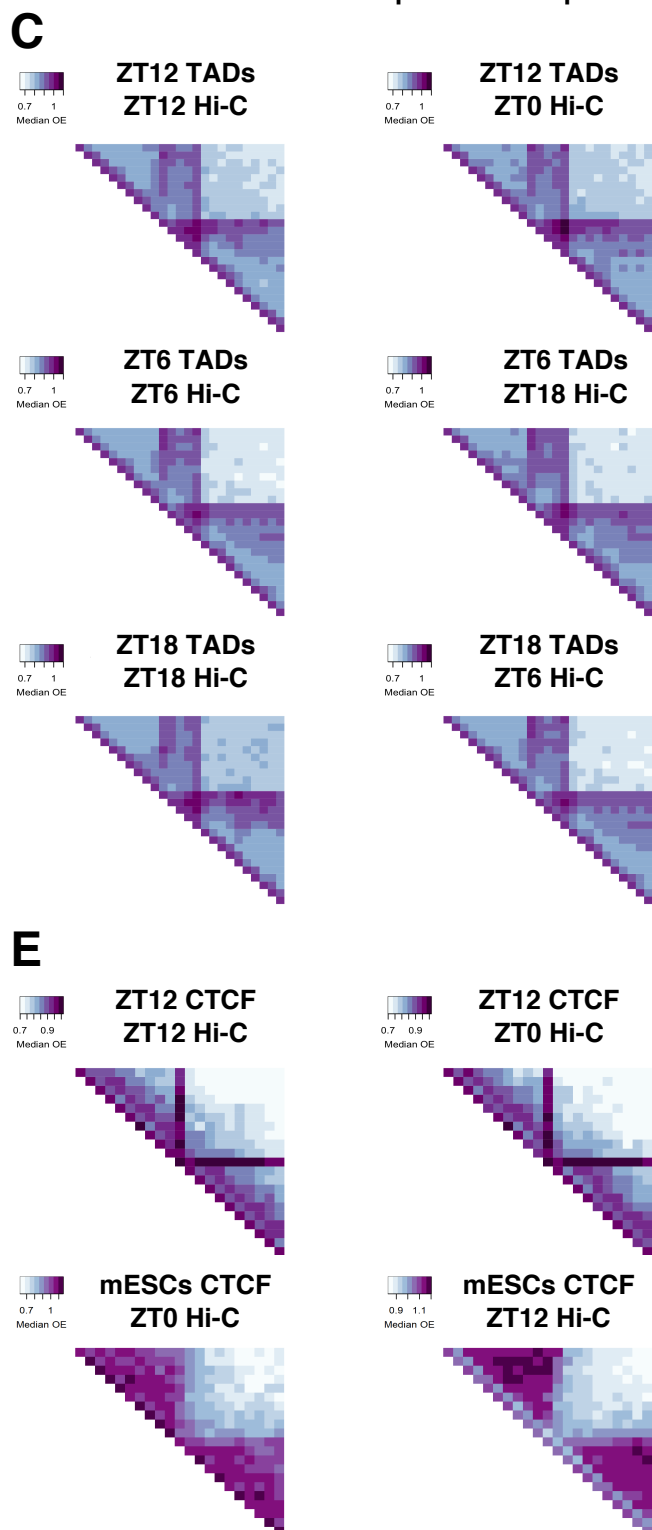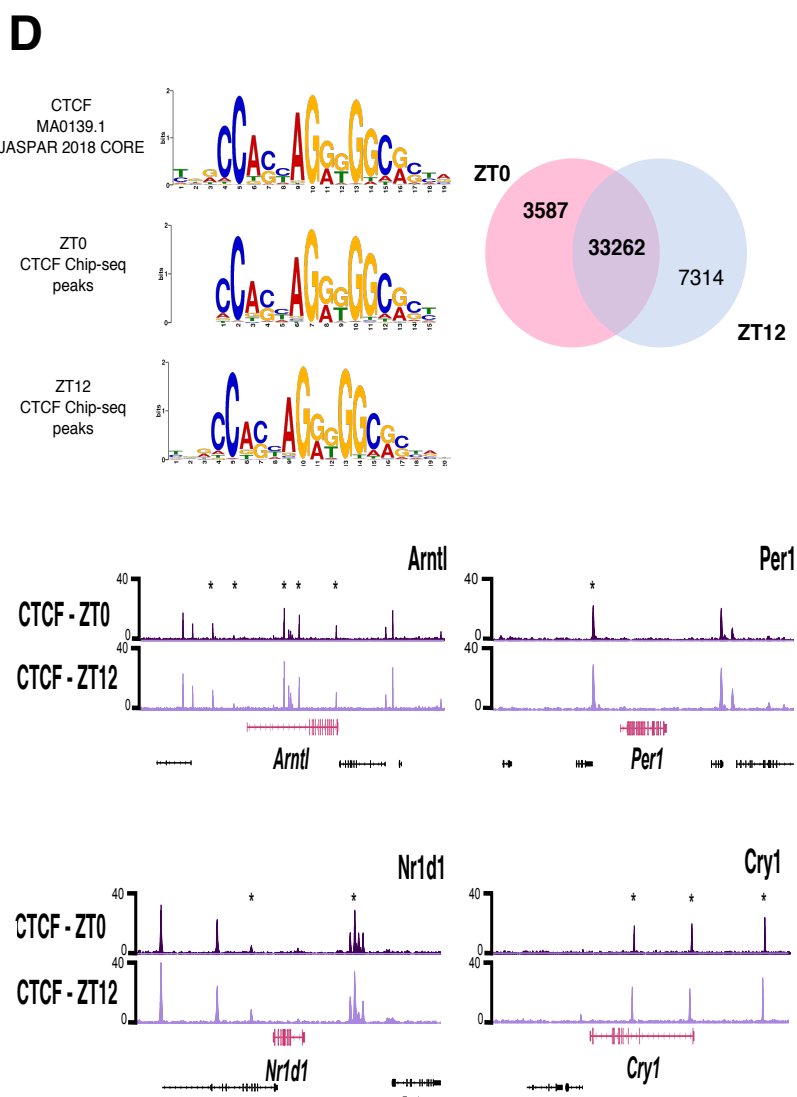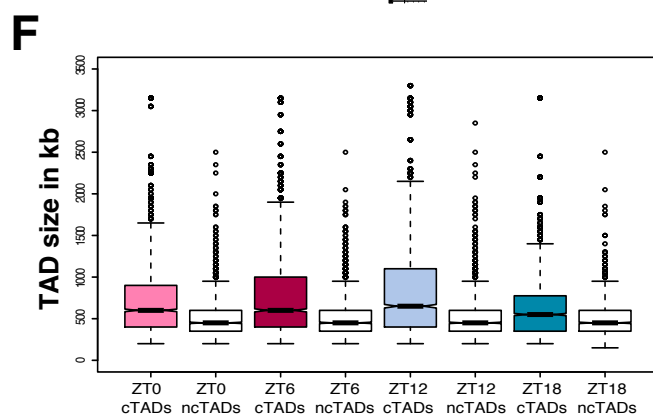

Supplement: Supplementary file 4 — Additional file 4: Figure S4. TAD structure and CTCF binding is preserved during the 24 hours. A. TADs overlap between time points. B. TADs size distribution at all time points. C. 50kb resolution Median Observed/Expected Hi-C signal around 1000 randomly selected TADs from ZT6, 12 and 18 plotted on the indicated time points. TADs were scaled to fit the five central bins. D. CTCF ChIP-seq motif analysis and peak overlap between ZT0 and 12. Genomic tracks of CTCF ChIP-seq signal at example regions harbouring circadian genes. E. Above, the same metaplots as in C but for 1000 randomly CTCF peaks found at ZT12 plotted using ZT12 and ZT0 Hi-C contacts. Below, metaplot using CTCF peaks from mESC and plotted using liver ZT0 and 12 Hi-C contacts. CTCF peaks are at the central bin of the metaplot. F. TAD and cTAD size comparison (p-value < 2.2e-16, Wilcoxon rank sum test). [file 13059_2021_2374_MOESM4_ESM.pdf]

**A**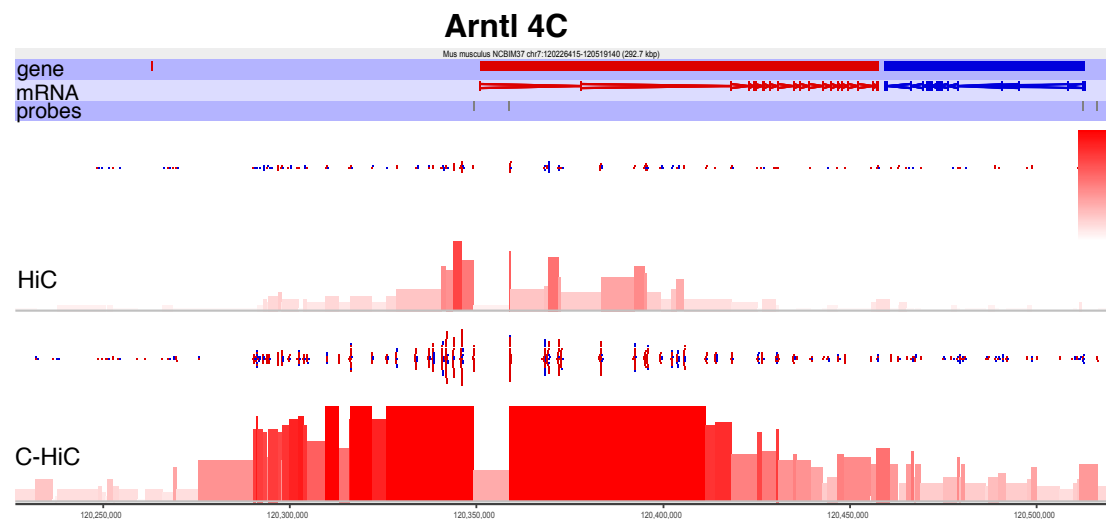**B**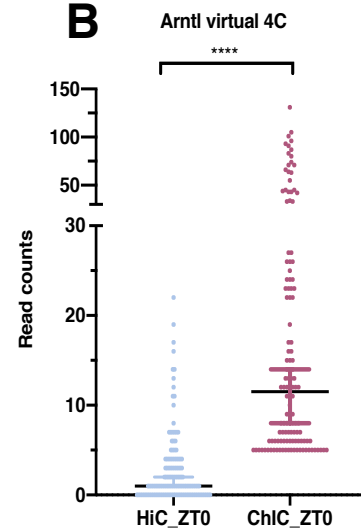**C****ZT0**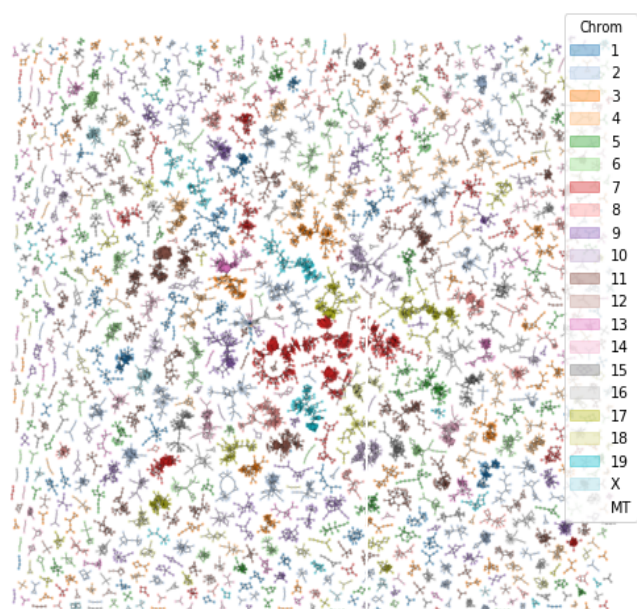**D****ZT0****ZT6****ZT12****ZT18**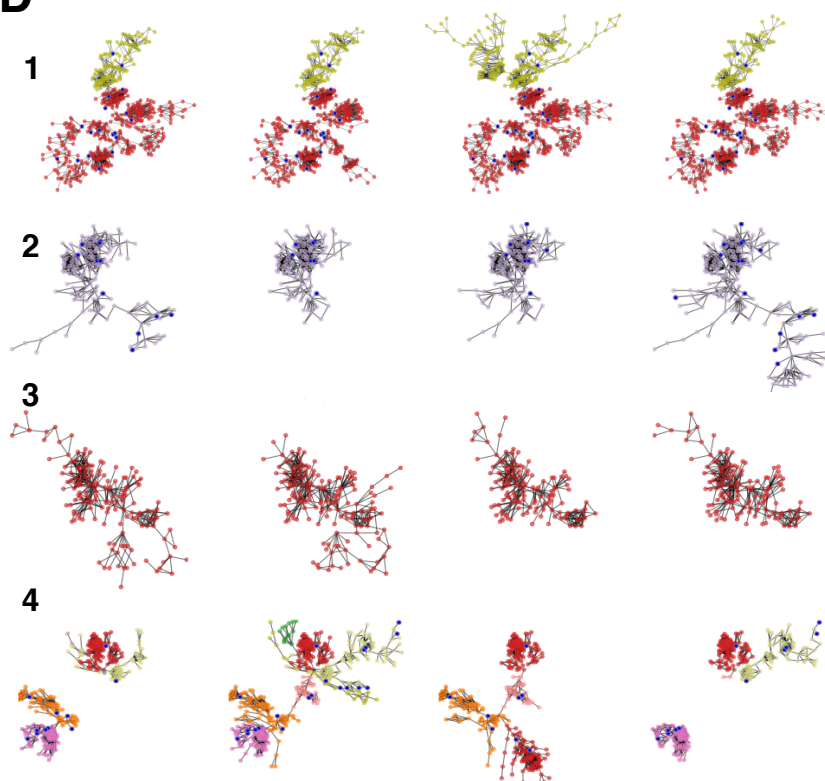**E****1**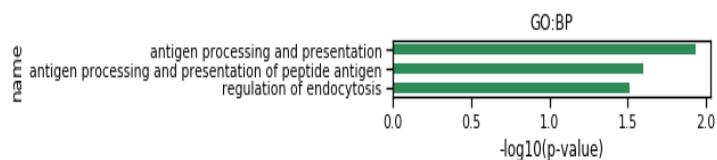**3**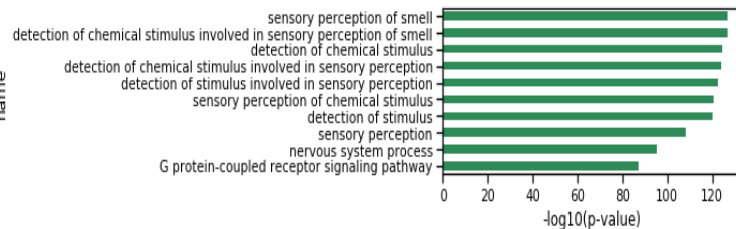**2**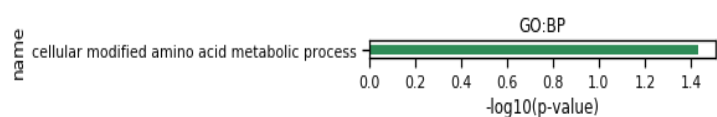**4**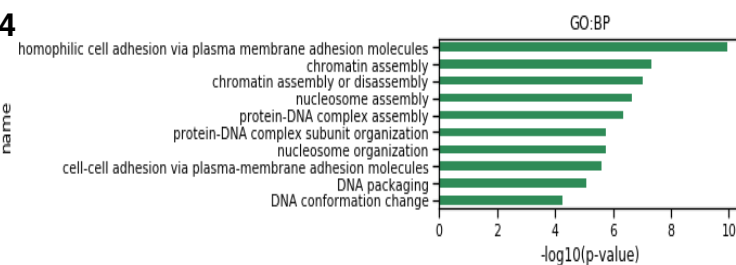

Supplement: Supplementary file 5 — Additional file 5: Figure S5. Promoter-Promoter networks in the mouse liver over a circadian cycle. A. Partial view of a virtual 4C from the Arntl gene promoter using the Hi-C and P-CHi-C raw valid pairs. Histograms of read counts per restriction fragment around the bait region corresponding to the captured promoter are shown. B. Number of total read counts comparing Hi-C and P-CHi-C recovered using the Arntl1 gene promoter as bait on a virtual 4C (restriction fragments with at least 5 reads in the P-CHi-C experiment where used) (p-value < 0.0001, Wilcoxon ranked test). C. Promoter-promoter contact network at ZT0. Each color represents a chromosome. Nodes with degree=1 are not included for simplicity. D. The four largest promoter-promoter interaction clusters of the network at the different time points during the day. E. Significantly enriched GO categories for the genes on the most prominent promoter-promoter clusters shown in D. [file 13059_2021_2374_MOESM5_ESM.pdf]

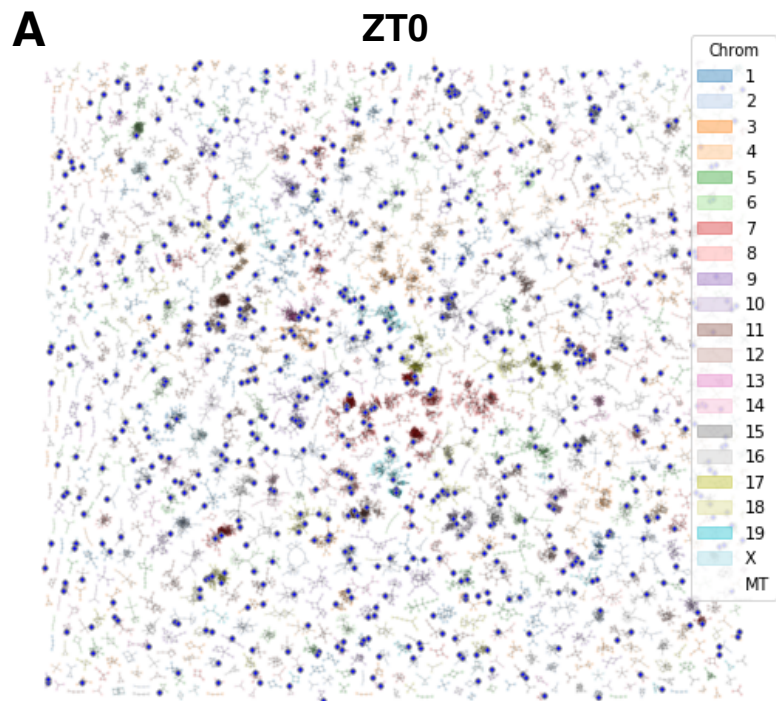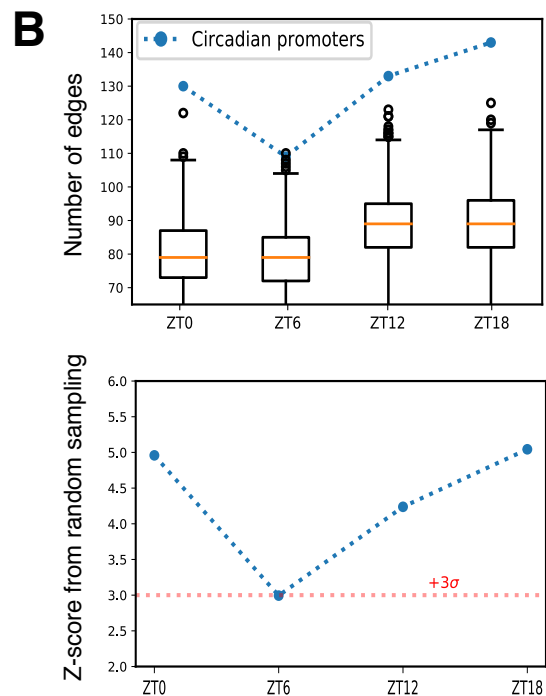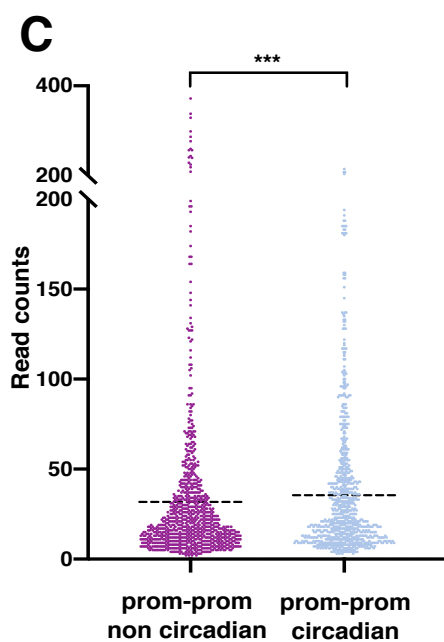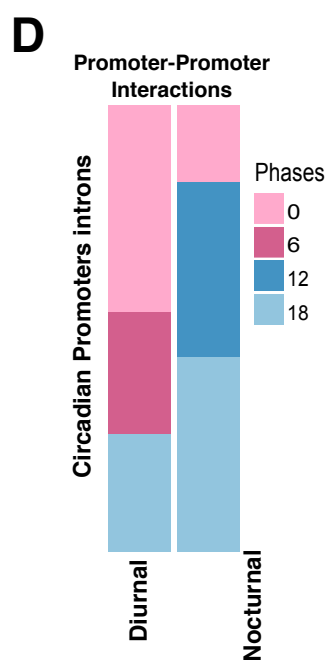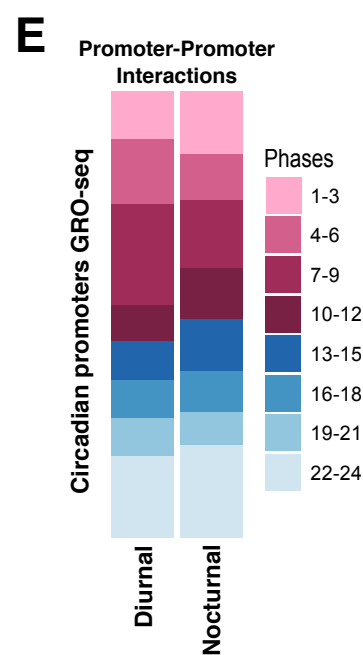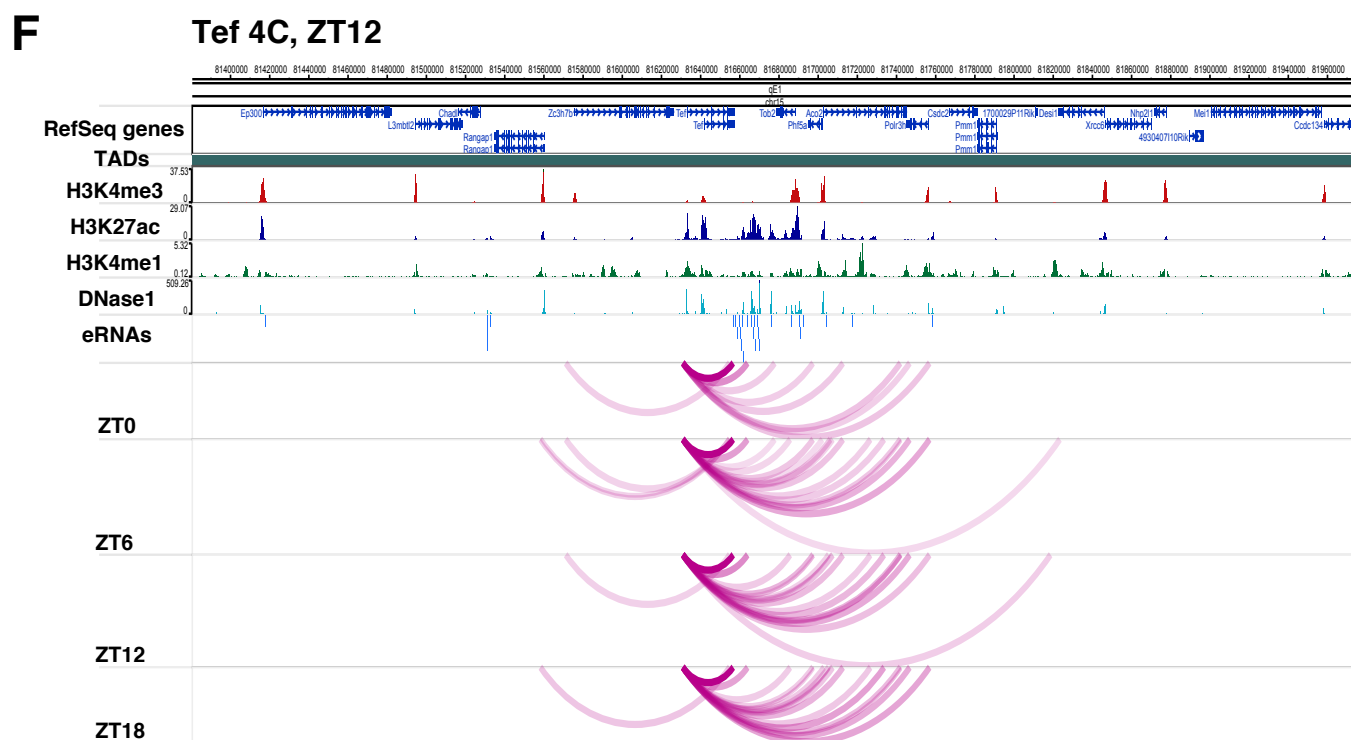

Supplement: Supplementary file 6 — Additional file 6: Figure S6. Circadian gene promoter-promoter interactions. A. Promoter-promoter contact network at ZT0 with the circadian genes marked in blue. B. Above, number of edges (interactions) between circadian gene promoters at ZT0, 6, 12 and 18 hours of the day (blue line) compared to a random set of non circadian promoters. Below, z-score for circadian gene promoter contacts compared to the random sampling. C. Quantification of the read counts supporting all circadian gene promoter-promoter interactions compared to a random set of non circadian promoter-promoter interactions (*** p-value < 0.001, Mann Whitney test) D. Transcriptional phase distributions of circadian promoters in contact with diurnal or nocturnal circadian promoters (all p-values < 0.0001, Wilcoxon signed rank test) for our circadian intronic gene set. E. The same as in D for the circadian gene set detected through GRO-seq (Fang et al., 2014) (all p-values < 0.0001, Wilcoxon signed rank test) F. Virtual 4C landscape for the Tef circadian gene promoter from P-CHi-C data at all time points during the day. The acrophase of Tef is written next to the gene name. Expression profiles for both genes can be found in Figure S3. Genomic tracks show significant contacts as arcs and chromatin features including liver H3K4me3, H4K4me1, H3K27ac, DNAseI, eRNAs and TADs. Tef gene promoter contacts Aco2 gene promoter both with acrophase at ZT12. [file 13059_2021_2374_MOESM6_ESM.pdf]

## A Rorc 4C, ZT18

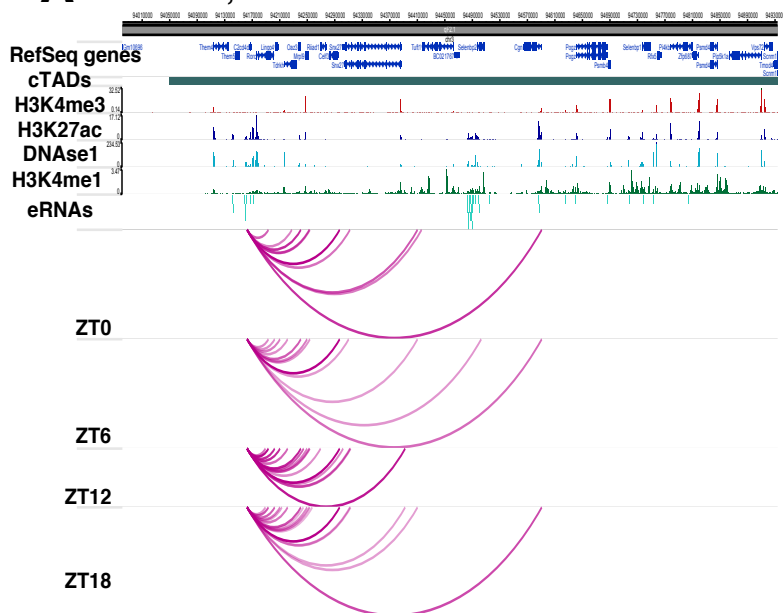

## B Npas2 4C, ZT0

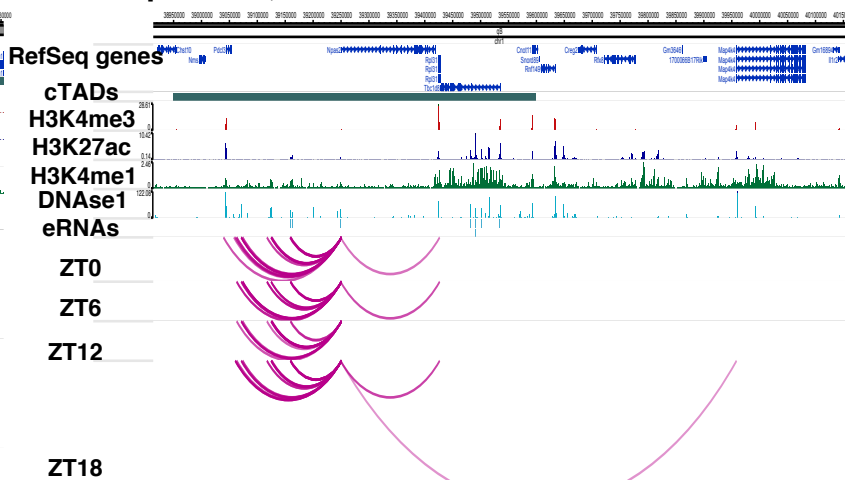

## C Nr1d2 4C, ZT12

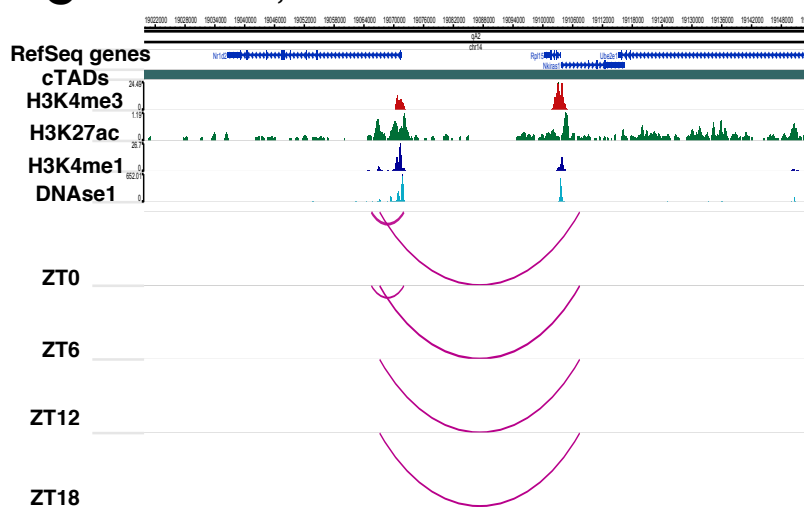

## D Per2 4C, ZT18

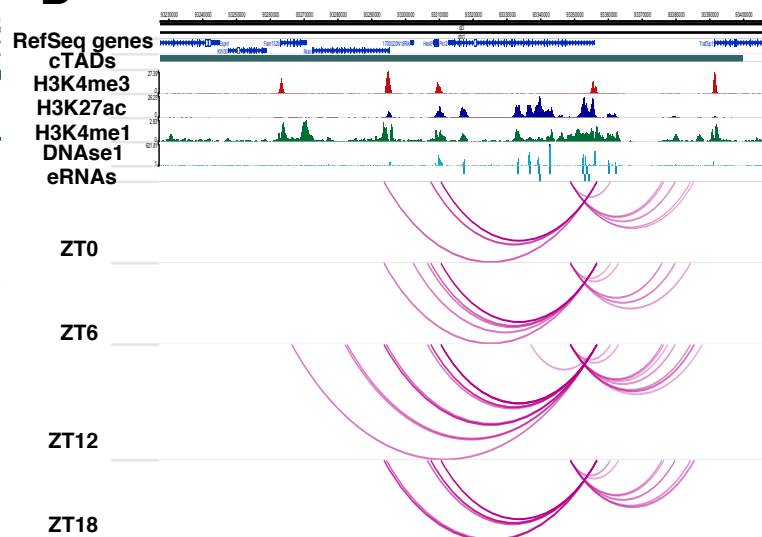

## E Dhr3 4C, ZT0

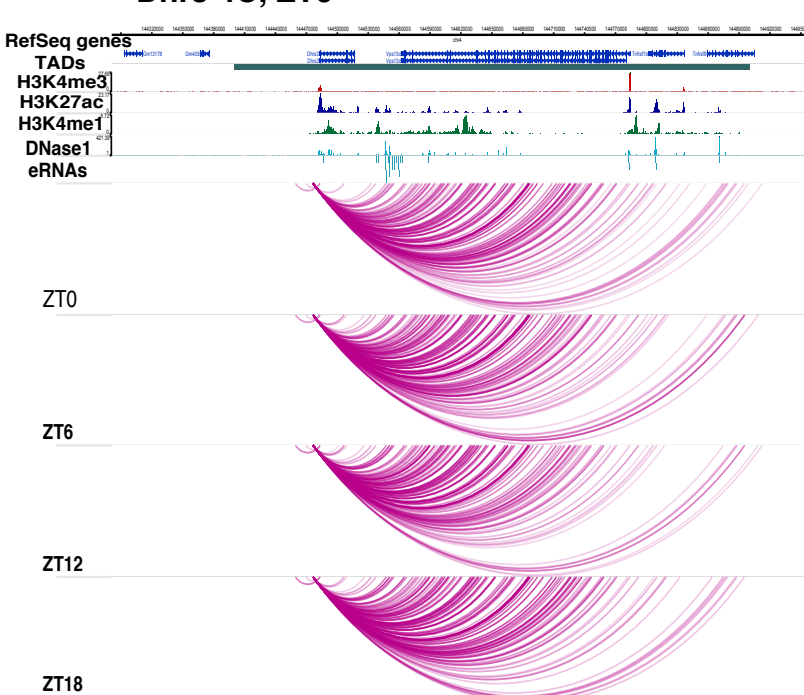

## F Ppp1r3c 4C, ZT0

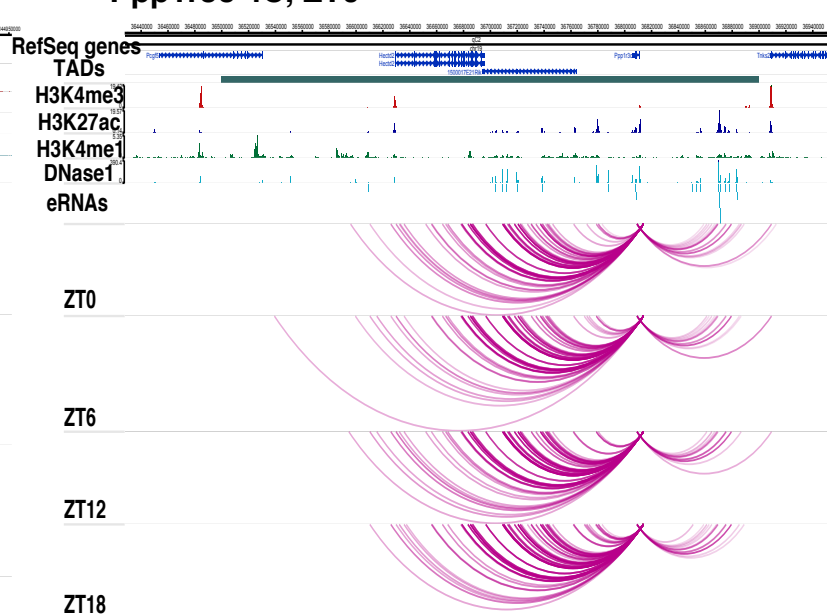

Supplement: Supplementary file 7 — Additional file 7: Figure S7. Core clock gene promoter vs output circadian genes interaction landscapes.A-D. Virtual 4C for Rorc, Npas2, Nr1d2 and Per2, core clock circadian gene promoters from P-CHi-C data at all time points during the day. The Rorc circadian gene promoter contacts Cgn circadian gene promoter and both peak in transcription at ZT18. E-F Virtual 4C for Dhrs3 and Ppp1r3c output circadian genes in the liver. Acrophases are written next to the gene names. Genomic tracks show significant contacts as arcs and chromatin features including liver H3K4me3, H3K4me1, H3K27ac, DNAseI, eRNAs and TADs. Expression profiles for all genes can be found in Figure S3. The interaction profiles of core clock genes display less contacts that dynamically change over time. The two output gene contact profiles show more saturated contacts that are mostly constant during the 24 hours. [file 13059_2021_2374_MOESM7_ESM.pdf]
