# Supplementary material for: The global and promoter-centric 3D genome organization temporally resolved during a circadian cycle
Source: Genome Biol. 2021 Jun 8;22:162. doi: 10.1186/s13059-021-02374-3 (PMC8185950; doi:10.1186/s13059-021-02374-3)
Supplement: Supplementary file 9 — Additional file 9. Review history. [file 13059_2021_2374_MOESM9_ESM.docx]

**Review history**

**First round of review**

**Reviewer 1**

In liver hundreds to thousands of genes are transcribed in a circadian/diurnal fashion. This subject is covered by an extensive literature on circadian transcriptomics (both with regard to nascent transcripts and mature mRNAs), promoter and enhancer screening, chromatin modifications (e.g. histone marks) and chromatin topology. In their paper Furlan-Magaril et al. focus on the latter subject, specifically on the reversible transitions in chromatin contacts accompanying oscillatory transcription. To this end they used an arsenal of currently use state-of-the-art genomics methods, including Hi-C, Promoter Capture (i.e. RNA driven hybrid enrichment of promoter-containing fragments from Hi-C libraries), ChIP-seq (using antibodies for CTCF, a chromatin boundary/insulating factor), and the data-mining tools required to quantitatively analyze the large data sets generated by these technologies. Studies with similar aims and outcomes have already been published by the groups of Felix Naef using 4C (PMID:  29572261; PMID:  29254942) and Mitch Lazar using 3C and hi-C (PMID:  29439026). In brief, Furlan-Magaril confirmed several conclusions made by the authors of these previously published studies. In particular, their results revealed transitions in chromatin contacts that correlate with the acro- and nadir phases of transcription, the invariable demarcations of TADs across the 24-hour day, and the cooperation of clock transcription factors(TFs) with liver-enriched TFs (such as NR5A2) in driving hepatocyte-specific diurnal transcription. In keeping with the latter finding, core clock genes establish fewer contacts than clock-controlled genes, presumably because the promoters of the latter also interact with liver-specific enhancers (or enhancers occupied by signal-sensing TFs). What are the major novelties of the study?  First, chromosome capture experiments were conducted at a higher resolution than that achieved in previous studies, and second, the hi-C library was screened for promoter-containing fragments, which provided important insights in promoter-enhancer interactions. In addition, the authors identified an unexpectedly high proportion (17%) of TADs that undergo transitions between open and closed chromatin configurations.
Clearly, the study cannot be classified as "paradigm changing" (a term that is, admittedly , overused and abused). Nonetheless, I find the findings reported by Furlan-Magaril interesting and worthy of publication. Yes, some of the reported data are confirmative rather than novel. However, chromosome conformation capture technologies (and in particular hi-C) are highly artifact-prone - a lesson I learned from the inventor (Job Dekker) himself. Therefore, it is important that results obtained with these approaches are verified independently in multiple laboratories.
I must emphasize, however, that I am not an expert in bioinformatics and that I can thus not evaluate the data mining procedures employed by the authors with due rigor. Here the editors will have to rely on the comments/criticisms made by referees who are more competent in the bioinformatic analysis of large data sets.
Specific comments:
(1) How do the contact regions relate to Dnase 1-hypersensitive sites (DHSS), which in liver were mapped genome-wide around the clock (PMID: 28414715)? One would expect that the restriction fragments encompassing promoter and/or enhancer sequences would contain DHSS. This should be discussed for a few diurnally expressed genes.
(2) Lines 38-41. The sentence "Individual circadian gene promoters displayed maximal chromatin contacts at times of peak transcriptional output and the expression of circadian genes and contacted transcribed regulatory elements, or other circadian genes, was phase-coherent" is incomprehensible. It should be subdivided into smaller bits.  
(3) Lines 122-137. The terms PCA (principal component analysis) and PC1n and how they are used in genomics should be explained in a few sentences (even though the authors provide a reference for this).
(4) Lines 143-145. The authors considered genes to be cyclically expressed, when at least one pair of time points were statistically different. This will probably yield a large number of false positives. There are many studies published by other authors that provide genome-wide circadian transcriptome data (RNA-seq and microarray data) with a much higher temporal resolution. The authors should examine whether the 1257 transcripts reported on line 145 are indeed expressed in a circadian fashion. This can be readily done by consulting John Hogenesch's circaDB database http://circadb.hogeneschlab.org/.
(5) Lines 197-199. The authors should indicate that mESCs do not harbor functional circadian oscillators (PMID:  20133594). Hence, the delimitation of TADs encompassing circadian genes by CTCF and other factors are established before these genes are rhythmically expressed. This should be mentioned.
(6) Line206-209. Figure 2D is not mentioned in the text (unless I missed something).
(7) Citations are very sloppy, indicating that the paper has not been proofread carefully before submission. Make sure that each citation is listed under "References", and that each reference appearing under "References" is referred to in the text.
Examples
Line 344. The reference (Balsalobre et al., 1998) is not listed under "References".
Lines 822-823. The reference Damiola et al. is not mentioned in the text. Moreover, the citation is false and incomplete. The authors probably refer to Balsalobre et al., 1998 (PMID: 9635423).
Lines 835-836. The reference Elphege N (2017) is incomplete and should read Nora E.P, etc (PMID:  28525758).
Lines 855-857. Check spelling of reference (Hagberg hagberg….)
Lines 878-879. https://doi.org/10.1126/science.aao6891 is repeated twice.
Line 897-899. Check reference!

**Reviewer 2**

In their paper, Furlan-Magaril et al. use chromosome conformation capture technology to investigate 3D genome folding dynamics during the circadian cycle. Obtaining data at 4 timepoints, they identify regions of dynamics compartment switching during the circadian cycle, as well as TADs that harbour multiple circadian genes that appear to be co-regulated. Promoter-centric Hi-C analysis indicated 3D interactions between circadian genes with similar expression profiles. Binding motifs for transcription factors were identified at stable and dynamic interaction anchors involving circadian genes. Finally, the authors make the observation that core clock genes tend to have fewer interacting elements but show more dynamic interaction profiles.    

The datasets provided in this paper are of high quality and will likely serve as an interesting resource in the field. Overall, the analyses performed with these data are strictly descriptive and feel somewhat rushed, as does the writing and presentation of the paper. The authors do make several interesting observations, in particular regarding A/B compartment dynamics and circadian gene co-regulation in TADs.  

Major comments:
- Figure 1G and 1H are discussed after Fig.2, please correct order in the figures. In fact, this happens so often in the paper that I have a very hard time reading the manuscript. I urge the authors to double-check whether the order of discussing the data matches the order in which the figures show the data.
- In their analysis of A/B compartment dynamics, the authors solely focus on those bins that change sign (i.e. switch). This strategy doesn't consider large changes in A/B compartment association that do not result in a compartment switch, which could be biologically meaningful (PMID: 29335546, 33299181). If no strong argument against including non-switching dynamic bins exists, I suggest the authors expand their compartment analysis along those lines.
- Fig.S5. The authors use a rather complicated network modeling approach to determine that circadian genes interact more often with each other. While the analyses they present seem valid (although difficult to fully grasp), why have they not directly quantified a normalized interaction strength/frequency between circadian genes vs. non-circadian genes? It would make their claim a lot more solid (and this paper of the paper much easier to follow). Another method would be to use their Hi-C data and perform aggregate peak analysis (PMID: 25497547) using the promoters of circadian and random/non-circadian genes.
- The examples used to illustrate synchronized circadian transcription between interacting circadian genes (e.g. Fig.S4F, Fig.S5G, Fig.4E-G) do not show any gene expression data across the circadian cycle - which makes them rather poor examples to illustrate this point... The authors need to include both pCHI-C and expression data.
- It is completely unclear to me what Fig.S4D-E and Fig.S5F show, as the authors fly over this analysis in the text and the legend. What is meant here with a phase distribution? How do these plots show that diurnal and nocturnal genes are in close proximity? This part needs to be made much more accessible.
- Fig.3G. The authors identify novel putative transcription factors that controls dynamic looping at circadian genes, which is interesting. However, without any attempt to validate these findings this part of the paper remains very speculative. Novelty would significantly benefit from a focused validation effort to show that these factors are indeed important for circadian gene control.

Minor comments:
- Line 156, typo: 'Kluskal'
- Fig.1C/D. Unclear what exactly is shown here. Are these log2 fold changes or was some sort of normalisation used to obtain the negative log2 RPM values?
- No reference to Fig.2D?
- Fig.2F. What is the % of random TADs that overlap with OCCs? The text states the difference is statistically significant, but it's important to quantify the magnitude of the increase here.
- Many figures look messy and need to be formatted properly to improve readability of the paper (e.g. font sizes that vary widely between two panels of the same figure). Especially the use of very small font sizes should be avoided.

**Authors’ response to reviewers**

Reviewer reports:

Reviewer #1: In liver hundreds to thousands of genes are transcribed in a circadian/diurnal fashion. This subject is covered by an extensive literature on circadian transcriptomics (both with regard to nascent transcripts and mature mRNAs), promoter and enhancer screening, chromatin modifications (e.g. histone marks) and chromatin topology. In their paper Furlan-Magaril et al. focus on the latter subject, specifically on the reversible transitions in chromatin contacts accompanying oscillatory transcription. To this end they used an arsenal of currently use state-of-the-art genomics methods, including Hi-C, Promoter Capture (i.e. RNA driven hybrid enrichment of promoter-containing fragments from Hi-C libraries), ChIP-seq (using antibodies for CTCF, a chromatin boundary/insulating factor), and the data-mining tools required to quantitatively analyze the large data sets generated by these technologies. Studies with similar aims and outcomes have already been published by the groups of Felix Naef using 4C (PMID:  29572261; PMID:  29254942) and Mitch Lazar using 3C and hi-C (PMID:  29439026). In brief, Furlan-Magaril confirmed several conclusions made by the authors of these previously published studies. In particular, their results revealed transitions in chromatin contacts that correlate with the acro- and nadir phases of transcription, the invariable demarcations of TADs across the 24-hour day, and the cooperation of clock transcription factors(TFs) with liver-enriched TFs (such as NR5A2) in driving hepatocyte-specific diurnal transcription. In keeping with the latter finding, core clock genes establish fewer contacts than clock-controlled genes, presumably because the promoters of the latter also interact with liver-specific enhancers (or enhancers occupied by signal-sensing TFs). What are the major novelties of the study?  First, chromosome capture experiments were conducted at a higher resolution than that achieved in previous studies, and
second, the hi-C library was screened for promoter-containing fragments, which provided important insights in promoter-enhancer interactions. In addition, the authors identified an unexpectedly high proportion (17%) of TADs that undergo transitions between open and closed chromatin configurations.
Clearly, the study cannot be classified as "paradigm changing" (a term that is, admittedly , overused and abused). Nonetheless, I find the findings reported by Furlan-Magaril interesting and worthy of publication. Yes, some of the reported data are confirmative rather than novel. However, chromosome conformation capture technologies (and in particular hi-C) are highly artifact-prone - a lesson I learned from the inventor (Job Dekker) himself. Therefore, it is important that results obtained with these approaches are verified independently in multiple laboratories.
I must emphasize, however, that I am not an expert in bioinformatics and that I can thus not evaluate the data mining procedures employed by the authors with due rigor. Here the editors will have to rely on the comments/criticisms made by referees who are more competent in the bioinformatic analysis of large data sets.

Specific comments:

(1) How do the contact regions relate to Dnase 1-hypersensitive sites (DHSS), which in liver were mapped genome-wide around the clock (PMID: 28414715)? One would expect that the restriction fragments encompassing promoter and/or enhancer sequences would contain DHSS. This should be discussed for a few diurnally expressed genes

We thank the reviewer for his/her suggestion. We have now calculated the enrichment of the DHSS reported in PMID: 28414715 on the promoter interacting regions and included it in the results section for all promoter interacting regions (Figure 3B, left) and for circadian gene promoters (Figure 3C, right). We found that DHSS are enriched at promoter interacting regions and even more so at circadian gene promoter interacting regions. For simplicity we also add the results in this letter (Figure R1).

**Figure R1. DNase1 hypersensitive sites (DHS) are enriched in promoter interacting regions**. Above. DHS are enriched at all gene promoter interacting regions (p-value < 4.286069e-250, t test). Below. DHS are enriched at circadian gene promoter interacting regions, both the full set of circadian genes and circadian genes oscillating in transcription at the intronic level (see methods) (p-value < 2.259606e-130 and 1.835416e-137, t test).

(2) Lines 38-41. The sentence "Individual circadian gene promoters displayed maximal chromatin contacts at times of peak transcriptional output and the expression of circadian genes and contacted transcribed regulatory elements, or other circadian genes, was phase-coherent" is incomprehensible. It should be subdivided into smaller bits.

We thank the reviewer for his/her observation rightly pointing out the complicated wording. We have now modified the paragraph as follows:

“ We found circadian gene promoters displayed a maximal number of chromatin contacts at the time of their peak transcriptional output. Furthermore, circadian genes as well as contacted and transcribed regulatory elements reached maximal expression at the same time points.”

(3) Lines 122-137. The terms PCA (principal component analysis) and PC1n and how they are used in genomics should be explained in a few sentences (even though the authors provide a reference for this).

We have now added the following phrase to the manuscript to clarify the meaning of the PCA analysis: “PCA analysis is used to analyse high dimensional data and redefine it with as few dimensions as possible that explain most of the variance in the data. As such, the 1st principal component (PC1) will explain the majority of the variance, followed by the second component (PC2) and so forth. When applying PCA analysis to the normalized contact Hi-C matrix from individual chromosomes, important features can be identified. For most chromosomes the PC1 value reflects two distinct interaction compartments that correspond to open and closed chromatin (Lieberman-Aiden et al., 2009)”

(4) Lines 143-145. The authors considered genes to be cyclically expressed, when at least one pair of time points were statistically different. This will probably yield a large number of false positives. There are many studies published by other authors that provide genome-wide circadian transcriptome data (RNA-seq and microarray data) with a much higher temporal resolution. The authors should examine whether the 1257 transcripts reported on line 145 are indeed expressed in a circadian fashion. This can be readily done by consulting John Hogenesch's circaDB database <http://circadb.hogeneschlab.org/>.

We thank the reviewer for his/her suggestion. We have calculated the overlap between our circadian gene set and two other data sets with higher temporal resolution available in the literature and extensively used (Koike et al., 2012 and Fang et al., 2014) (Figure R2). The overlap between our circadian gene set and the exonic (Figure R2A) and intronic (Figure R2B) gene set from Koike et al 2012 is 37 and 22% respectively. The overlap between our circadian gene set and the genes detected as rhythmic in Fang et al 2014 is 20% (Figure R2C). The overlap between the circadian genes detected in Koike et al 2012 (exonic set) and Fang et al., 2014 is 15% and 28% transcribed genes, respectively (Figure R2D). When comparing the overlap between the three data sets (considering the exonic circadian gene set from Koike et al., 2012) we find 45% of our gene set being shared with one or the other circadian gene data sets, which is a higher proportion than the one observed for any of the other two published circadian gene sets (38% for Koike and 32% for Fang) (Figure R2E). Finally, we filtered out the circadian transcripts identified in this study without an overlap with the exonic set from Koike et al., 2012 from the 4 time points and analysed them in circaDB (<http://circadb.hogeneschlab.org/>), as suggested. As shown in Figure R2F, 75%-81% of our circadian gene set is readily detectable as circadian in CircaDB. Examples of our total RNA-seq signal are shown for genes without an overlap with Koike et al., 2012 but included in circaDB (Figure R2F lower panel). All of these results show that in general, there is limited overlap between the circadian genes detected in different studies and through different methods. However the list of genes categorized as circadian in this study have the highest overlap with either of the data sets analysed and is also confirmed as rhythmic in the circaDB. In addition both Gene Ontology analysis and KEGG analysis confirmed the efficient recovery of circadian gene functional categories describing circadian genes from our total RNA-seq as presented in the manuscript. Importantly, throughout the manuscript we have presented data for an intronic circadian gene set which was obtained by matching our circadian genes with the intronic circadian gene set from Koike et al., 2012, or with the circadian gene set detected through GRO-seq by Fang et al., 2014 (Figures 3B,C, 4B,C and D, S6D and E), which confirms our claims in these independent circadian gene sets.

**Figure R2.** **Overlap between our circadian gene set and three published data sets.** A. Overlap between our circadian gene set and Koike et al., 2012 circadian genes, exon set. B. Overlap between our circadian gene set and Koike et al., 2012 circadian genes, intron set. C. Overlap between our circadian gene set and Fang et al., 2014 circadian gene set. D. Overlap between Koike et al., 2012 (exonic set) and Fang et al., 2014 circadian gene sets. E. Overlap between the three data sets. F. Upper panel. Proportion of our circadian gene set that do not overlap with Koike et al., 2012 circadian gene set and is foundas rhythmic in CircaDB. Lower panel. Total RNA-seq signal of circadian genes present in our data set and found in CircaDB.

(5) Lines 197-199. The authors should indicate that mESCs do not harbor functional circadian oscillators (PMID:  20133594). Hence, the delimitation of TADs encompassing circadian genes by CTCF and other factors are established before these genes are rhythmically expressed. This should be mentioned.

We thank the reviewer for his/her suggestion. We did not assess the conservation of TADs between ESCs and liver, only CTCF chromatin occupancy in the two model systems. Thus we have now added to the manuscript: “The conservation of CTCF binding between ESCs and adult liver tissue shows that the insulation of circadian genes by CTCF precedes the onset of their rhythmic transcription, as ESCs do not harbour functional circadian oscillators (Yagita et al., 2010).”

(6) Line206-209. Figure 2D is not mentioned in the text (unless I missed something).

We have now modified line 209 as follows: (Figure 2D and E, only data from cTADs with 2 circadian genes shown).

(7) Citations are very sloppy, indicating that the paper has not been proofread carefully before submission. Make sure that each citation is listed under "References", and that each reference appearing under "References" is referred to in the text.

Examples
Line 344. The reference (Balsalobre et al., 1998) is not listed under "References".
Lines 822-823. The reference Damiola et al. is not mentioned in the text. Moreover, the citation is false and incomplete. The authors probably refer to Balsalobre et al., 1998 (PMID: 9635423).
Lines 835-836. The reference Elphege N (2017) is incomplete and should read Nora E.P, etc (PMID:  28525758).
Lines 855-857. Check spelling of reference (Hagberg hagberg….)
Lines 878-879. <https://doi.org/10.1126/science.aao6891> is repeated twice.
Line 897-899. Check reference!

We apologize and thank the reviewer for his/her comment and the thorough revision of the references. We have now carefully check the references and corrected all the mistakes as pointed out by the reviewer. Indeed the Damiola et al., reference was incorrect and refers to Balsalobre et al., 1998.

Reviewer #2: In their paper, Furlan-Magaril et al. use chromosome conformation capture technology to investigate 3D genome folding dynamics during the circadian cycle. Obtaining data at 4 timepoints, they identify regions of dynamics compartment switching during the circadian cycle, as well as TADs that harbour multiple circadian genes that appear to be co-regulated. Promoter-centric Hi-C analysis indicated 3D interactions between circadian genes with similar expression profiles. Binding motifs for transcription factors were identified at stable and dynamic interaction anchors involving circadian genes. Finally, the authors make the observation that core clock genes tend to have fewer interacting elements but show more dynamic interaction profiles.     

The datasets provided in this paper are of high quality and will likely serve as an interesting resource in the field. Overall, the analyses performed with these data are strictly descriptive and feel somewhat rushed, as does the writing and presentation of the paper. The authors do make several interesting observations, in particular regarding A/B compartment dynamics and circadian gene co-regulation in TADs.

Major comments:
- Figure 1G and 1H are discussed after Fig.2, please correct order in the figures. In fact, this happens so often in the paper that I have a very hard time reading the manuscript. I urge the authors to double-check whether the order of discussing the data matches the order in which the figures show the data.

We thank the reviewer for his/her comment. We have now carefully gone through the manuscript and organized the order of the figures according to when we mention them in the text. All changes in the manuscript are visible using the track changes tool.

- In their analysis of A/B compartment dynamics, the authors solely focus on those bins that change sign (i.e. switch). This strategy doesn't consider large changes in A/B compartment association that do not result in a compartment switch, which could be biologically meaningful (PMID: 29335546, 33299181). If no strong argument against including non-switching dynamic bins exists, I suggest the authors expand their compartment analysis along those lines.

We thank the reviewer for her/his comments. Initially, we focused on the analysis of bins that switch compartments during the 24 hour-cycle (Oscillatory Chromatin Compartments, OCCs), as we expected to detect robust changes in transcription and chromatin modifications in these regions across time points. As suggested by the reviewer, we have now extended the analysis to explore the bins with the most variability in PC1 values from both intra compartments A (positive PC1 values), and B (negative PC1 values) as described in (PMID: 29335546). For the intra-compartmental analysis we took the compartments called at 100 Kb that did not change PC1 sign in the four timepoints. To detect the highest variance between timepoints we calculated the standard deviation, then divided the bins into quartiles where the first quartile represents the least variation and the fourth quartile the highest variation of PC1 value across timepoints (Figure R3A,B). We focused on the fourth quartile bins for further analysis. First, we measured the overlap between TADs containing at least one circadian gene (cTADs) and the fourth quartile bins compared to the bins corresponding to OCCs as reported in the manuscript. We found 40% (341) of cTADs overlapping with bins in the fourth quartile for the A compartments, 25% (214) of cTADs overlapping with bins in the fourth quartile of the B compartments and 73% (623) of cTADs overlapping with OCCs as reported in the manuscript (Figure R3C). From these, the overlap between cTADs and OCCs and A variable compartments is significantly more than expected for a random set of the same number of domains and is less than expected for bins in the most variable B compartments. Interestingly 246 out of 341 (72%) cTADs overlapping with the most variable A compartments also overlap with OCCs, thus cTADs tend to include both bins changing compartment assignments and bins fluctuating within the A compartment during the day.

Next we measured the RNA content in fourth quartile bins from both the A and B compartments. We did not detect significant changes in RNA content between them across time points (Figure R3D,E). Finally we filtered the bins from the fourth quartile from A and B compartments by the highest and lowest PC1 per timepoint (i.e. bins with maximum or minimum PC1 value at ZT0, ZT6 and so on) (Figure R3F, only bins with maximum and minimum PC1 values at ZT0 and 12 shown), and measured their RNA content across time points. As shown for ZT0 and ZT12 we did not detected significant differences in RNA content between time points for these bins in contrast with what we observed for OCCs as reported in the manuscript (Figure 1G). These results suggest that, in comparison with systems in which there are considerable changes in transcription in a large proportion of genes, for instance during cell differentiation and development (PMID: 29335546), our system represents a fairly constant transcriptional environment with only a small proportion of genes with rhythmic expression corresponding to circadian genes (~10% of the transcriptome). Thus, the bins that change compartment during the 24 hours represent the most relevant category reflecting significant changes in transcription in this time frame.

**Figure R3. Analysis of intra-compartment PC1 variable regions**. A. Standard Deviation of PC1 values between timepoints for intra-compartment regions. The fourth quartile represents the regions with the most variable PC1 values within A or B compartments. B. PC1 values of the four quartiles calculated in A across timepoints. C. Overlap of circadian TADs with regions from the 4^th^ quartile A, B and Oscillatory Chromatin Compartments (OCCs) compared to a random control (*** p < 0.0001, t-test). D. RNA content analysis (RPM, Reads Per Million) in the intra compartments variable regions from A and E. B compartments. No significant changes are detected between timepoints (one-way ANOVA test). F. PC1 values for intra compartment variable regions with maximum and minimum PC1 values at ZT0 and ZT12. G. RNA content analysis (RPM, Reads Per Million) in the intra compartments variable regions with maximum and minimum PC1 values at ZT0 and ZT12. No significant changes are detected between timepoints (one-way ANOVA test).

- Fig.S5. The authors use a rather complicated network modeling approach to determine that circadian genes interact more often with each other. While the analyses they present seem valid (although difficult to fully grasp), why have they not directly quantified a normalized interaction strength/frequency between circadian genes vs. non-circadian genes? It would make their claim a lot more solid (and this paper of the paper much easier to follow). Another method would be to use their Hi-C data and perform aggregate peak analysis (PMID: 25497547) using the promoters of circadian and random/non-circadian genes.

We thank the reviewer for his/her suggestion. The analysis we have done from the network analysis presented in now Figure S6B only counts the number of interactions (edges) between circadian promoters compared to a random set of non-circadian promoters showing how the former, form significantly more contacts than the control. As suggested by the reviewer, we have now looked at the number of reads supporting circadian promoter-promoter interactions compared to a random set of non circadian promoter-promoter interactions and added it as Figure S6C and here in Figure R4 for simplicity. The results show that circadian promoter-promoter contacts are more robust compared to non-circadian promoter-promoter contacts (Figure R4, left). The original figure 5SF (now Figure 56D) shows how the circadian promoter pairs involved in an interaction tend to share the transcriptional acrophase, with diurnal circadian promoters contacting other diurnal circadian promoters and nocturnal circadian promoters contacting other nocturnal circadian promoters. Thus, in summary we found that circadian promoter-promoter contacts are more abundant and more robust than non circadian promoter-promoter contacts and that circadian promoter pairs involved in an interaction tend to share their transcriptional acrophase.

We have added the following text to the manuscript to describe this new result as follows:

“Next we examined the circadian component in promoter-promoter networks. Circadian promoters are dispersed across chromosomes (Figure S6A, blue dots represent circadian gene promoters). Nevertheless, circadian promoters establish significantly more contacts among them compared to non-circadian gene promoters (Figure S6B. Above, comparison of the number of edges formed between circadian promoters compared to a random set of non-circadian gene promoters. Below, Z-scores compared to the random sampling of non-circadian promoters). Next we looked at the reads supporting significant interactions between circadian gene promoters in contrasts with interactions between non circadian gene promoters. The result shows that circadian promoter-promoter contacts are more robust compared to non circadian promoter-promoter contacts (Figure S6C, p-values < 0.001, Mann-Whitney test). Finally, we compared the time of maximal mRNA abundance of circadian genes whose promoters were contacting each other. To do this we separated the circadian gene promoters into diurnal and nocturnal based on their transcriptional acrophase and then analysed the time of maximal RNA expression of their contacted circadian gene promoters. We found that diurnal promoters preferentially contact other diurnal genes. Likewise, nocturnal circadian genes preferentially contact other genes with nocturnal expression maxima (Figure S6D, our intronic circadian gene set, p-values < 0.0001 Wilcoxon signed rank test). Similar results were obtained for circadian genes detected by GROseq (Fang et al., 2014) reflecting primary transcription (Figure S6E, p-values < 0.0001 Wilcoxon signed rank test). This is exemplified by the interaction between the *Tef* and the *Aco* circadian gene promoters with shared pre-messenger expression peak at ZT12 (Figure S6F) as well as the interaction between *Rorc* and *Cgn* circadian genes with shared acrophase at ZT18 (Figure S7A). Thus, in summary the results show that circadian promoter-promoter contacts are more robust than non circadian promoter-promoter contacts. Furthermore, pairs of interacting circadian gene promoters tend to share their transcriptional acrophase.”

As suggested by the reviewer we also generated aggregate peak analysis for both circadian promoter-promoter contacts and the non circadian control, however the differences are not evident in this representation of the data (Figure R4, right).

**Figure R4. Circadian gene promoter-promoter interactions are more robust than non circadian gene promoter-promoter interactions.** Left. Read counts supporting circadian promoter-promoter contacts in comparison to a random set of non circadian promoter-promoter interactions (*** p-value <0.001, Mann-Whitney test). Right, aggregate peak analysis of circadian and non circadian promoter-promoter interactions.

- The examples used to illustrate synchronized circadian transcription between interacting circadian genes (e.g. Fig.S4F, Fig.S5G, Fig.4E-G) do not show any gene expression data across the circadian cycle - which makes them rather poor examples to illustrate this point... The authors need to include both pCHI-C and expression data.

In each figure we indicate the acrophase of the circadian gene exemplified in the title at the top of the corresponding panel. As suggested by the reviewer we have now included the RNA-seq signal for the circadian gene presented in all examples across the paper as an additional Supplementary Figure (Figure S3). We did not included the RNA-seq tracks for all timepoints directly into the WashU genome browser as the genomic scale used to visualize the interactions does not allow for clear visualisation of the rhythmic gene expression of the circadian genes presented.

- It is completely unclear to me what Fig.S4D-E and Fig.S5F show, as the authors fly over this analysis in the text and the legend. What is meant here with a phase distribution? How do these plots show that diurnal and nocturnal genes are in close proximity? This part needs to be made much more accessible.

We thank the reviewer for his/her suggestion. The plots presented in Figure S4D (now Figure 4D), analyse the transcriptional acrophase of circadian genes and their interacting enhancer elements. Figure S4E and S5F (now Figure S6D and E) analyse the transcriptional acrophase of circadian genes interacting with other circadian genes. To do these analyses we separated the circadian gene promoters into diurnal and nocturnal depending on their time of transcriptional peak or acrophase and analysed the time of transcription or acrophase of the contacted enhancers or circadian gene promoters.

We have now modified the text in the manuscript to explain these analyses more clearly as follows:

“Finally, we compared the time of maximal mRNA abundance of circadian genes whose promoters were contacting each other. To do this we separated the circadian gene promoters into diurnal and nocturnal based on their transcriptional acrophase and then analysed the time of maximal RNA expression of their contacted circadian gene promoters. We found that diurnal promoters preferentially contacted other diurnal genes. Likewise, nocturnal circadian genes preferentially contacted other genes with nocturnal expression maxima (Figure S6D, our intronic circadian gene set, p-values < 0.0001 Wilcoxon signed rank test). Similar results were obtained for circadian genes detected by GROseq (Fang et al., 2014) reflecting primary transcription (Figure S6E, p-values < 0.0001 Wilcoxon signed rank test).”

For the new Figure 4D we explain the following: “We then compared the transcriptional phases between promoters of circadian genes and their corresponding contacted enhancer elements with rhythmic transcription. To do so we separated the circadian gene promoters into diurnal and nocturnal depending on their transcriptional acrophase and then analysed the time of maximal RNA expression of their contacted enhancer elements. We found a significant contact preference between diurnal promoters and diurnal enhancers as well as nocturnal promoters and nocturnal enhancers (Figure 4D, left). These preferences were more pronounced when analysing our circadian intronic gene set reflecting primary transcription (see methods) (Figure 4D, centre) or detected by GRO-seq (Figure 4D, right, all p-values < 0.0001 Wilcoxon signed rank test).”

- Fig.3G. The authors identify novel putative transcription factors that controls dynamic looping at circadian genes, which is interesting. However, without any attempt to validate these findings this part of the paper remains very speculative. Novelty would significantly benefit from a focused validation effort to show that these factors are indeed important for circadian gene control.

We thank the reviewer´s suggestion. To this end we have performed Chromatin Immunoprecipitation (ChIP-qPCR) experiments for the liver nuclear receptor Nr5a2 and quantified its enrichment at the four time points during the day for three loci in which the DNA binding motif was found enriched. The results have been included in Figure 3G of the manuscript and as Figure R5 for simplicity in this letter.

We found Nr5a2 dynamically occupying the *Arntl* circadian gene promoter with peak occupancy at ZT6 when *Arntl* gene expression starts to decrease. The same, but less pronounced, binding pattern over time was observed on the *Arntl* interacting enhancer (Figure R5). We then analysed Nr5a2 binding to a chromatin region stably contacting the circadian gene promoter of *Ppp1r3c*. Interestingly, we found that Nr5a2 binding was dynamic with maximum occupancy at ZT6 when *Ppp1r3c* gene expression starts to decrease (Figure R5). In addition we also assessed Nr5a2 occupancy in a chromatin region dynamically interacting with the circadian gene promoter of *Gsk3a* but did not find the receptor bound to this region (Figure R5). Thus our results suggest that Nr5a2 is dynamically occupying regulatory elements of circadian genes irrespective of whether the contacts to circadian gene promoters are constant or rhythmic over time.

Experiments to test the functional importance of Nr5a2, and other candidates, in the circadian regulation of chromatin loop formation and cyclic gene expression have been initiated and are being performed in the laboratory. However, a thorough execution and analysis of loss-of-function and other experiments will unlikely be at hand in a meaningful time span for this manuscript and will be published at a later timepoint.

**Figure R5. Nr5a2 chromatin occupancy during the 24 hours**. ChIP-qPCR experiments were performed at four time points during the 24 hours in 2 biological replicates. The regions analysed include the Arntl gene promoter and dynamically contacted enhancer, a constant interacition with the Ppp1r3c circadian gene and a dynamic interaction of the Gsk3a circadian gene. In all regions the DNA binding motif for Nr5a2 was found. As a negative control a region without the motif was used. (* p-value < 0.05, ** p-value < 0.001, *** p-value < 0.0001, One-way ANOVA, Tukey posthoc test, n=6 from 2 biological replicates).

Minor comments:
- Line 156, typo: 'Kluskal'

We have replaced “Kluskal” with “Kruskal”

- Fig.1C/D. Unclear what exactly is shown here. Are these log2 fold changes or was some sort of normalisation used to obtain the negative log2 RPM values?

The plots presented in Figure 1C and D show the log2 Reads Per Million (RPM) quantification of the total RNA-seq reads in all the A and B compartments in the four timepoints (C) and in the regions changing compartments during the 24 hours (Oscillatory Chromatin Compartments, OCCs) in D. Given than the RPM measure can be fractional, the log2 values can be negative. For instance if there are 2 reads over a region and we had 10 million reads in the library, the RPM value would be 0.2, thus the log2 results in -2.32, a negative value.

- No reference to Fig.2D?

We have now modified line 209 as follows: (Figure 2D and E, only data from cTADs with 2 circadian genes shown).

- Fig.2F. What is the % of random TADs that overlap with OCCs? The text states the difference is statistically significant, but it's important to quantify the magnitude of the increase here.

We thank the reviewer for the suggestion. We have now replaced the graph in Figure 2F to show the magnitude of the increase instead of the percentage.

- Many figures look messy and need to be formatted properly to improve readability of the paper (e.g. font sizes that vary widely between two panels of the same figure). Especially the use of very small font sizes should be avoided

We have gone through the figures and modified the font sizes for them to be readable.

**Second round of review**

**Reviewer 1**

In the revised version of their manuscript the authors have appropriately addressed all of my concerns and comments. I therefore feel that this interesting paper is now fit for publication.

**Reviewer 2**

The authors did a very good job in revising the paper according to my comments and those made by the other reviewer. I support publication of this interesting manuscript.
